# Characterization of unique B-cell populations in the circulation of people living with HIV prior to non-Hodgkin lymphoma diagnosis

**DOI:** 10.3389/fimmu.2024.1441994

**Published:** 2024-09-11

**Authors:** Laura E. Martínez, Begoña Comin-Anduix, Miriam Güemes-Aragon, Javier Ibarrondo, Roger Detels, Matthew J. Mimiaga, Marta Epeldegui

**Affiliations:** ^1^ UCLA AIDS Institute, University of California, Los Angeles, Los Angeles, CA, United States; ^2^ Department of Obstetrics and Gynecology, David Geffen School of Medicine, University of California, Los Angeles, Los Angeles, CA, United States; ^3^ Jonsson Comprehensive Cancer Center, University of California, Los Angeles, Los Angeles, CA, United States; ^4^ Ahmanson Translational Theranostics Division, Department of Molecular and Medical Pharmacology, University of California, Los Angeles, Los Angeles, CA, United States; ^5^ Division of Surgical Oncology, Department of Surgery, University of California, Los Angeles, Los Angeles, CA, United States; ^6^ Department of Hematology and Oncology, David Geffen School of Medicine, University of California, Los Angeles, Los Angeles, CA, United States; ^7^ Department of Epidemiology, Fielding School of Public Health, University of California, Los Angeles, Los Angeles, CA, United States

**Keywords:** mass cytometry, B-cells, B regulatory cells, HIV+ cART-naïve, HIV+ pre-NHL (cART-naïve)

## Abstract

People living with HIV (PLWH) are at higher risk of developing lymphoma. In this study, we performed cytometry by time-of-flight (CyTOF) on peripheral blood mononuclear cells of cART-naïve HIV+ individuals and cART-naïve HIV+ individuals prior to AIDS-associated non-Hodgkin lymphoma (pre-NHL) diagnosis. Participants were enrolled in the Los Angeles site of the MACS/WIHS Combined Cohort Study (MWCCS). Uniform Manifold Approximation and Projection (UMAP) and unsupervised clustering analysis were performed to identify differences in the expression of B-cell activation markers and/or oncogenic markers associated with lymphomagenesis. CD10^+^CD27^-^ B cells, CD20^+^CD27^-^ B cells, and B-cell populations with aberrant features (CD20^+^CD27^+^CXCR4^+^CD71^+^ B cells and CD20^+^CXCR4^+^cMYC^+^ B cells) were significantly elevated in HIV+ cART-naïve compared to HIV-negative samples. CD20^+^CD27^+^CD24^+^CXCR4^+^CXCR5^+^ B cells, CD20^+^CD27^+^CD10^+^CD24^+^CXCR4^+^cMYC^+^ B cells, and a cluster of CD20^+^CXCR4^hi^CD27^-^CD24^+^CXCR5^+^CD40^+^CD4^+^AICDA^+^ B cells were significantly elevated in HIV+ pre-NHL (cART-naïve) compared to HIV+ cART-naïve samples. A potentially clonal cluster of CD20^+^CXCR4^+^CXCR5^+^cMYC^+^AICDA^+^ B cells and a cluster of germinal center B-cell-like cells (CD19^-^CD20^+^CXCR4^+^Bcl-6^+^PD-L1^+^cMYC^+^) were also found in the circulation of HIV+ pre-NHL (cART-naïve) samples. Moreover, significantly elevated clusters of CD19^+^CD24^hi^CD38^hi^ cMYC^+^ AICDA^+^ B regulatory cells were identified in HIV+ pre-NHL (cART-naïve) compared to HIV+ cART-naïve samples. The present study identifies unique B-cell subsets in PLWH with potential pre-malignant features that may contribute to the development of pre-tumor B cells in PLWH and that may play a role in lymphomagenesis.

## 1 Introduction

B cells are a crucial component of humoral immunity and the adaptive immune system. B cells are specialized in antigen presentation, antibody production, immune memory, and class switching, and promote regulatory functions (1). B cells can be classified into various subsets by their marker expression. Common B-cell subsets include naïve B cells (CD19^+^CD20^+^CD27^-^), IgM-expressing memory B cells (CD19^+^CD20^+^CD27^+^IgM^+^), class-switched memory B cells (CD19^+^CD20^+^CD27^+^IgG/IgA), activated B cells (CD19^+^CD20^+^CD38^+^ B cells), germinal center B cells involved in affinity maturation and class switching (CD19^+^CD20^+^CXCR5^+^Bcl-6^+^), terminally differentiated B cells that secrete antibodies or plasma cells (CD19^+/-^CD27^++^CD38^++^CD24^-^CD138^+^IgD^-^), and regulatory B cells involved in immune regulation and suppressing inflammation (CD19^+^CD24^+^CD38^+^) (1–5).

CD24 plays key roles in B-cell development and activation. CD24 is expressed during early B-cell development in the bone marrow and is upregulated on activated B cells (1, 6). The expression of CD24 in germinal center B cells aids in the selection and maturation of B cells undergoing affinity maturation.

CD40 is a co-stimulatory molecule expressed on B cells as it plays a vital role in their activation, differentiation, and function. CD40 interacts with CD40 ligand (CD40L) on T-helper cells to promote B-cell activation and proliferation in response to antigen encounter (7). CD40 signaling is critical for class switching, enabling B cells to produce different antibody isotypes (e.g., switching from IgM to IgG, IgA, or IgE) in order to develop effective and specific responses against pathogens. CD40 is also important for the formation and maintenance of germinal centers in lymphoid tissues where B cells undergo affinity maturation and selection. Other crucial co-stimulatory molecules expressed on B cells are CD80 (B7-1) and CD86 (B7-2), which play a role in immune activation and regulation. Binding of CD80/CD86 to CD28 on T cells enhances T-cell activation, proliferation, and survival. Engagement of CD80/CD86 promotes class switching in B cells and provides survival signals to activated B cells to promote long-lived immune responses (1, 6).

CD10 is an early B-cell marker involved in signaling pathways that promote B-cell activation, proliferation, and differentiation (8, 9). CD10 is also commonly expressed on germinal center B cells, influencing their differentiation into memory B cells or plasma cells (9). Moreover, CD10 is a classical marker in the diagnosis of certain B-cell neoplasms, including B-cell non-Hodgkin lymphoma (NHL) (10).

The risk of B-cell non-Hodgkin lymphoma (NHL) is increased in chronic HIV infection (11). Although combination anti-retroviral therapy (cART) has improved the overall survival of persons living with HIV, NHL remains a significant cause of morbidity and mortality among HIV+ individuals after the introduction of cART (11). Chronic HIV infection leads to immune suppression, which can allow for the uncontrolled growth of abnormal cells and increase the risk of developing different types of lymphomas, such as Burkitt lymphoma (BL), diffuse large B-cell lymphoma (DLBCL), primary central nervous system lymphoma (PCNSL), plasmablastic lymphoma (PBL), and primary effusion lymphoma (PEL) (11–13). People living with HIV (PLWH) may develop lymphomas that are heterogeneous in nature due to different pathogenic mechanisms that include chronic exposure to antigen, genetic mutations, dysregulation and production of pro-inflammatory cytokines, and the loss of immunoregulation of oncogenic viruses [i.e., Epstein-Barr Virus (EBV), Kaposi’s sarcoma-associated herpesvirus (KSHV)] (11, 13–16).

The presence of pre-malignant B cells are likely to drive the development of lymphoma. HIV-related chronic immune activation, inflammation, and the immune responses governing the pre-tumor microenvironment are likely to allow the initiating stages of lymphoma. Aberrant B cells are elevated in the circulation of HIV+ individuals, including elevated fractions of CD10^+^ and CD71^+^ B cells (17). Other work corroborated these findings by showing that B cells expressing CD10, CD71, or CD86 are elevated in those who went on to develop AIDS-NHL (18). Chronic B-cell activation may also lead to the expression of activation-induced cytidine deaminase (AICDA), a DNA-modifying enzyme. AICDA is mainly expressed in germinal center (GC) B cells and induces immunoglobulin gene class switch recombination (CSR) and somatic hypermutation (SHM) in GC B cells (19). Chronic AICDA expression can lead to lymphomagenic molecular lesions, oncogenic translocation, and oncogenic mutations (20, 21). Induction of AICDA leads to the accumulation of genomic uracil in B-cell lymphoma cell lines and unique mutational signatures in human B-cell malignancies (22). In addition, AICDA gene expression is elevated in circulating B cells of HIV+ subjects before an AIDS-NHL diagnosis, and specifically in those who go on to develop Burkitt’s lymphoma (23). We have shown that CD40L incorporated into HIV virions induces B-cell activation and AICDA expression (24) and that EBV infection causes SHM events and the accrual of oncogenic mutations in B cells (25). The EBV latent membrane protein 1 (LMP1), which mimics CD40 signaling, can also induce AICDA expression (26).

Activated B cells may develop GC-like phenotypes associated with B-cell malignancies (10, 16, 27). CXCR4 and CXCR5 are two important chemokine receptors that mediate homing to secondary lymphoid tissues and that have been implicated in some of these B-cell malignancies, including NHL (28). CXCR4 is the chemokine receptor for SDF-1 (stromal cell-derived factor-1, also known as CXCL12) and is expressed in non-hematopoietic and hematopoietic cells, including several B-cell subsets, and with the highest levels on GC B cells (29). The interaction of CXCR4 with SDF-1 is important for the trafficking of lymphocytes and is essential for normal B-cell development as it is involved in the retention of B-cell precursors in the bone marrow and is associated with B-cell homing to lymph nodes (30). In the context of B-cell biology, CXCR5 is expressed by mature B cells in re-circulation (31). CXCR5 recruits circulating naïve B cells to follicles, and its ligand, CXCL13, is secreted by stromal cells in B-cell zones of secondary lymphoid follicles where they encounter antigen and differentiate (32).

We previously reported that B regulatory cells or Bregs (CD19^+^CD24^hi^CD38^hi^) are significantly elevated in HIV+ subjects and prior to an AIDS-NHL diagnosis, and that PD-L1-expressing B cells comprise a subpopulation of these Breg cells (33). Bregs comprise approximately 1% of circulating B cells (34), can suppress differentiation of pro-inflammatory lymphocytes, and promote the expansion of immunosuppressive T cells (4). The commonly ascribed functions of Bregs are immunosuppression and production of anti-inflammatory cytokines TGF-β and IL-10 (4). The immunoregulatory functions of Bregs are important as they can suppress HIV-1-specific CD8^+^ T cells’ responses (35). Thus, these unique and specialized B-cell subsets may contribute to the early stages and/or development of malignant pre-lymphoma B cells in NHL.

We and others have shown that elevated levels of several cytokines (IL-6, IL-10, CXCL13, TNFα, and IP-10/CXCL10) (14, 36, 37), molecules associated with B-cell activation (sCD23, sCD27, sCD30, and κ and λ immunoglobulin free light chains), and microbial translocation factors [e.g., lipopolysaccharide-binding proteins (LPB, sCD14, and EndoCab)] (38) precede the development of AIDS-associated NHL (AIDS-NHL). Serum levels of microbial translocation markers, including FABP2, LPS-binding protein (LBP), haptoglobin, sCD14, endotoxin core antibody (EndoCab) IgM, and markers of macrophage activation, such as sCD163, are associated with AIDS-NHL risk (38). In addition, pre-treatment plasma levels of biomarkers of immune activation/inflammation (sTNF-RII, sCD25), microbial translocation (sCD14), and/or macrophage activation (BAFF/BLyS and CCL2/MCP-1) are associated with overall survival and progression-free survival of AIDS-NHL patients (39).

In this study, we hypothesized that B cells display activated GC-like features and aberrant phenotypes in PLWH, and aberrant oncogenic features in PLWH prior to an AIDS-NHL diagnosis (HIV+ pre-NHL and cART-naïve). To identify pre-malignant B cells in the circulation of PLWH, we conducted deep phenotyping of B cells using a mass cytometry [cytometry by time-of-flight (CyTOF)] antibody panel composed of B7-family molecules, B-cell activation and differentiation markers, and oncogenic markers. We immunophenotyped B cells from stored archival peripheral blood mononuclear cells (PBMCs) isolated from persons who were HIV+ and cART-naïve who did not go on to develop NHL, as well as from HIV+ cART-naïve individuals who developed AIDS-NHL, in PBMCs collected prior to the diagnosis of NHL (HIV+ pre-NHL and cART-naïve) (median of 12 months), and who were participants in the Los Angeles site of the MACS/WIHS Combined Cohort Study (MWCCS). We compared B-cell phenotypes between HIV+ cART-naïve and HIV+ pre-NHL (cART-naïve) samples and also compared them with HIV-negative. We found that B-cell populations with aberrant features (CD20^+^CD27^+^CD10^+^CD24^+^CXCR4^+^cMYC^+^ B cells and CD20^+^CXCR4^hi^CD27^-^CD24^+^CXCR5^+^CD40^+^CD4^+^AICDA^+^ B cells) and Bregs expressing AICDA and cMYC were in the circulation of HIV+ pre-NHL (cART-naïve) compared to HIV+ cART-naïve individuals. The present study identifies B-cell subsets with potential pre-malignant phenotypes and oncogenic potential.

## 2 Materials and methods

### 2.1 Study participants

PBMCs were obtained from HIV-negative individuals (*n* = 10), HIV+ and cART-naïve individuals who did not develop NHL (*n* = 20), and HIV+ individuals who went on to develop NHL (*n* = 10) (HIV+ pre-NHL and cART-naïve). PBMCs were obtained from stored, viable cryopreserved PBMC vials maintained in a central repository of the UCLA site of the MWCCS. This study included only male study participants part of the Los Angeles site of the Multicenter AIDS Cohort Study (MACS). The MACS is a long-term prospective study of the natural and treated history of HIV infection and AIDS that began in 1984 that consists of adult gay and bisexual men from four MACS sites in metropolitan areas of the United States: Baltimore/Washington (John Hopkins University), Chicago (Northwestern University), Los Angeles (UCLA), and Pittsburgh (University of Pittsburgh). In 2019, the MACS merged with a large prospective HIV/AIDS study, the Women’s Interagency HIV Study (WIHS), to form the MACS/WIHS Combined Cohort Study, and throughout here, it is referred to as MWCCS. The PBMC samples used in this study were specifically from visits conducted by MACS study participants between 1985 and 2002. The MACS includes a large number of AIDS-related lymphoma cases (>200) and appropriate HIV-negative and HIV+ controls (40).

### 2.2 Ethics approval statement

The MWCCS was approved by the UCLA Institutional Review Board (IRB) to ensure safety and protection of participants involved in the human subjects research review committee (IRB# 20-002292); all participants provided written informed consent. All specimens and any clinical information provided by the MWCCS were de-identified.

### 2.3 Mass cytometry

#### 2.3.1 Mass cytometry antibody panel

The mass cytometry panel was created using the Maxpar Panel Designer software (Fluidigm/Standard BioTools) in which metal oxidation, antibody signaling, and antibody tolerance were taken into consideration. The CyTOF panel included surface antigen markers and cytokines to broadly immunophenotype B cells and their activation state: CD19, CD20, CD24, CD38, CD40, CD71, and HLA-DR. Markers of immune cell lineage were also included: T cells (CD3, CD4, and CD8) and monocytes (CD14, CD11b, and CD163). The panel included chemokine receptors for signaling (CXCR4, CXCR3, CXCR5, and CCR5), B-cell antibody secretion molecules (IgG, IgM, Ig kappa light chain, and Ig lambda light chain), checkpoint and signaling molecules (PD-1, PD-L1, PD-L2, ICOS, CD80, CD86, CD27, CD28, CD40L, and CTLA-4), transcription factors (Bcl-6 and FoxP3), an immunoregulatory cytokine (IL-10), oncogenic markers (CD10, cMYC, and AICDA), the EBV marker latent membrane protein-1 (LMP1), and a marker of active HIV infection (KC57). Pre-conjugated antibodies were purchased from Fluidigm/Standard BioTools or conjugated at the UCLA Jonsson Comprehensive Cancer Center (JCCC) and Center for AIDS Research Flow Cytometry Core Facility. Briefly, metal-isotope-labeled antibodies used in this study were conjugated using the MaxPar X8 Multimetal Labeling Kit for lanthanide metal isotopes and the MaxPar MCP9 Antibody Labeling Kit for cadmium metal isotopes, as per the manufacturer’s protocol (Fluidigm/Standard BioTools). Each conjugated antibody was quality checked and titrated to obtain optimal staining concentrations using PBMCs isolated from healthy blood donors obtained from the UCLA Virology Core and/or using B-cell lymphoma cell lines (Ramos, Raji, and 2F7). The extracellular and intracellular antibody panel used is summarized in **Supplementary Table 2**.

#### 2.3.2 Mass cytometry staining

Cryopreserved PBMCs were thawed into complete RPMI-1640 medium containing 10% FBS supplemented with 1% penicillin and streptomycin (Gibco) and recovered for 5 min at room temperature. PBMCs were then centrifuged for 5 min at 1,500 rpm at room temperature. Cell pellets were resuspended in 1 mL of complete RPMI and treated with 100 µL of DNase I (2.27 KU/mL, Sigma-Aldrich, Catalog No. D5025-150KU) and incubated for 30 min at 37°C in a 5% CO_2_ incubator. After the 30-min incubation, 9 mL of complete RPMI was added for a total of 10 mL, cells were resuspended, and an aliquot was taken to count cells. Cells were centrifuged for 5 min at 1,500 rpm at room temperature to wash out DNase I. Cell pellets were then resuspended in 1 mL of 5 µM Cell-ID Cisplatin (Fluidigm/Standard BioTools, Catalog No. 201064). The cisplatin was then quenched by adding Maxpar Cell Staining Buffer (MCSB) (Fluidigm/Standard BioTools, Catalog No. 201068) at 5× volume. Fc block (10% BSA in 1× PBS) was filter-sterilized and added to cells for a 10-min incubation at room temperature. The extracellular surface staining antibody cocktail (**Supplementary Table 2**) was added in MCSB and incubated at room temperature for 30 min. For intracellular staining, cells were resuspended in FoxP3 fixation/permeabilization solution (ThermoFisher Scientific, Catalog No. 00-5523-00) for 30 min at room temperature, washed with FoxP3 permeabilization buffer, and centrifuged at 800 × g for 5 min at room temperature. The intracellular antibody cocktail (**Supplementary Table 2**) was added and incubated for 1 h at room temperature. After washing, cells were resuspended in 1 mL of 200 nM iridium intercalation solution (Ir191/193) (Fluidigm/Standard BioTools, Cell-ID Intercalator-Ir-500 μM, Catalog No. 201192B) in MaxPar fix and perm buffer (Fluidigm/Standard BioTools, Catalog No. 201067) and incubated overnight at 4°C. The next day, cells were washed once in 1 mL of MCSB and twice in 1 mL of MilliQ ddH_2_O.

#### 2.3.3 Mass cytometry data acquisition

Immediately before acquisition, samples were filtered through a 35-μm nylon mesh cell strainer in MilliQ ddH_2_O. Cells were acquired at a rate of 400–600 events/s using a Helios Mass Cytometer (Fluidigm) at the UCLA JCCC and Center for AIDS Research Flow Cytometry Core Facility. Acquired data were pre-processed and normalized using an EQ four-element calibration bead-based normalization protocol in the CyTOF software. In the process of data acquisition, some samples were clogged in the Helios Mass Cytometer and needed to be re-run. Instead of concatenating the samples at the beginning of the acquisition process, those samples were concatenated later in the data analysis pipeline using the OMIQ data analysis platform (www.omiq.ai) (Dotmatics, Boston, MA, USA). Data were acquired from 10 HIV-negative PBMC samples (12 FCS data files), 20 HIV+ cART-naïve PBMC samples (22 FCS data files), and 10 HIV+ pre-NHL (cART-naïve) samples (11 FCS data files). All samples and data files were used in the analysis.

#### 2.3.4 Mass cytometry data analysis

Flow cytometry standard (FCS) files were uploaded into the OMIQ data analysis platform. After cytometry, calibration beads were eliminated and stabilization of time (191Ir+ vs. time) was evaluated to eliminate any anomalies in the spikes of time. Then, singlets (193Ir vs 191Ir) and viable single-cell events (194Pt+ events) were identified, and manual gating was performed to select CD14^-^CD11b^-^ cells. The cells were then sub-gated to identify CD3^+^ T cells and CD19^+^ B cells (**Supplementary Figure 1**). Manual gating of different B-cell populations was performed using OMIQ: CD19^+^ B cells, CD19^+^CD20^+^CXCR4^+^ B cells, CD19^+^CD20^+^CXCR4^hi^ B cells, CD19^-^CD20^+^ B cells, and Bregs (CD19^+^CD24^hi^CD38^hi^).

### 2.4 Dimensionality reduction using UMAP

For immune cell population analysis, total viable and single cells were subjected to unsupervised Uniform Manifold Approximation and Projection (UMAP) and clustering via FlowSOM algorithms using all markers in the panel (**Supplementary Table 2**). The FlowSOM results were examined as metaclusters using UMAPs and heatmaps in OMIQ. We optimized the clustering analysis by trial of different neighboring values (i.e., Neighbors = 8, 15, or 20) and FlowSOM x-dim and y-dim cluster values (x-dim and y-dim = 10 or 20) to identify the best conditions for separating and refining metaclusters. Once optimizing the UMAP and FlowSOM parameters, unsupervised clustering was rerun multiple times (3×) with new random seeds to confirm that similar populations were reproducibly found before proceeding to the refinement of the metacluster numbers, gating, and statistical comparisons.

### 2.5 Characterization of lineage cell populations

UMAPs were created in OMIQ from an equal subsampling of 100,000 viable cells for each group with the following settings: Neighbors = 20; Minimum Distance = 0.01; Components: 2; Metric = Euclidean; and Epochs = 200. FlowSOM was implemented using the following settings: x-dim = 20; y-dim = 20; rlen = 10; Distance metric = Euclidean.

### 2.6 Clustering of B cells using FlowSOM algorithms

For in-depth unsupervised analysis of different B-cell populations (CD19^+^ B cells, CD19^+^CD20^+^CXCR4^hi^ B cells, CD19^-^CD20^+^ B cells, or CD19^+^CD24^hi^CD38^hi^ Bregs), cells were separately subsampled (i.e., depending on the total number of cells available in the filter for analysis and they were equally distributed per cohort group) but processed by a similar UMAP and FlowSOM analysis workflow. For dimensionality reduction of CD19^+^ B cells, samples were downsampled to 75,000 cells per group [HIV-negative, HIV+ cART-naïve, and HIV+ pre-NHL (cART-naïve)]. For CD19^+^CD20^+^CXCR4^hi^ B cells, samples were downsampled to 12,500 cells per group comparison [HIV+ cART-naïve vs. HIV+ pre-NHL (cART-naïve)]. For CD19^-^CD20^+^ B cells, samples were downsampled to 20,000 cells per group comparison [HIV+ cART-naïve vs. HIV+ pre-NHL (cART-naïve)]. For Bregs (CD19^+^CD24^hi^CD38^hi^), samples were downsampled to a maximum of 5,000 available cells per group comparison [HIV+ cART-naïve vs. HIV+ pre-NHL (cART-naïve)]. For all B-cell populations mentioned above with the exception of CD19^-^CD20^+^ B cells, UMAPs were created in OMIQ with nearest neighbors set to 22 and the following settings: Minimum Distance = 0.01; Components: 2; Metric = Euclidean; and Epochs = 200, and cells were clustered by FlowSOM with x-dim = 18, y-dim = 18, rlen = 10, and a distance metric = Euclidean. For clearer plotting of CD19^-^CD20^+^ B cells, UMAPs were created in OMIQ with nearest neighbors set to 15 and the following settings: Minimum Distance = 0.01; Components: 2; Metric = Euclidean; and Epochs = 200. Cells were clustered by FlowSOM with x-dim = 15, y-dim = 15, rlen = 10, and a distance metric = Euclidean.

### 2.7 Statistics

Data were uploaded onto the OMIQ analysis platform. Manual gating or clustering algorithms (FlowSOM) were used to determine the frequency of individual cell populations. Differentially abundant B-cell populations were identified using a Mann–Whitney test (two-tailed and unpaired, non-parametric test) and/or the edgeR statistical program in OMIQ. OMIQ and GraphPad Prism 10.2.2 software was used for data analysis and graphic representations. For heatmaps, we compared median marker expression values between two groups using a Mann–Whitney test (two-tailed and unpaired, non-parametric test). Results were considered significantly different if the *p*-value was less than 0.05 (*p < 0.05; **p < 0.005; *** *p* < 0.0005). ns, not significant.

### 2.8 Data availability statement

The original contributions presented in the study are included in the article and **Supplementary Material**. Further inquiries can be directed to the corresponding author.

## 3 Results

### 3.1 MWCCS study participants

Study participants were cisgender men, and most were white, non-Hispanic (**Table 1**). All HIV+ (*n* = 20) and HIV+ pre-NHL samples (*n* = 10) were cART-naïve at study sample collection. PBMC samples from HIV+ pre-NHL (cART-naïve) were selected from visits prior to an AIDS-NHL diagnosis with a median of 12 months (range of 6 to 36 months). Information on the interval time between the sample used for CyTOF and the diagnosis of lymphoma is provided in **Supplementary Table 1**. Most cohort participants developed the DLBCL tumor subtype. The median age of each group was similar: HIV-negative (49 ± 5.6), HIV+ cART-naïve (44 ± 6.4), and HIV+ pre-NHL (cART-naïve) (44 ± 6.5) (**Table 1**). HIV-negative samples had overall higher median CD4^+^ T-cell counts (median count ± SD; 859 ± 309) compared to the HIV+ cART-naïve samples, while HIV+ pre-NHL (cART-naïve) had an overall higher median level of CD4^+^ T cells (775 ± 389) compared to HIV+ (579 ± 302). HIV-negative samples had overall lower median CD8^+^ T-cell counts (399 ± 461) compared to the HIV+ cART-naïve and HIV+ pre-NHL (cART-naïve) samples, while HIV+ cART-naïve had an overall higher median of CD8^+^ T cells (822 ± 512) compared to HIV+ pre-NHL (cART-naïve) (636 ± 403). In addition, ratios of CD4^+^/CD8^+^ T-cell counts were higher in HIV-negative (2.03 ± 0.64) compared to all HIV+ cART-naïve samples, but higher ratios were observed in HIV+ pre-NHL (cART-naïve) (1.04 ± 0.56) compared to HIV+ cART-naïve (0.71 ± 0.37) samples (**Table 1**). Information on viral load was limited. Standard viral load (copies/mL) was available for one HIV+ sample (67,931 copies/mL) and four HIV+ pre-NHL samples (median of 60,977 copies/mL; minimum of 8,547 copies/mL and maximum of 146, 095 copies/mL).

**Table 1:** Characteristics of MWCCS participants.

	HIV-negative	HIV+ (cART-naïve)	HIV+ pre-NHL (cART-naïve)
***N***	10	20	10
Age,			
**Median ± SD**	49 ± 5.6	44 ± 6.4	44 ± 6.5
**Min, age**	Min, 35	Min, 35	Min, 35
**Max, age**	Max, 54	Max, 56	Max, 56
Sex, Male			
***N* (%)**	100%	100%	100%
Race/Ethnicity, *N* (%)			
**White, non-Hispanic**	10 (100%)	19 (95%)	20 (100%)
**Black, non-Hispanic**	0 (0%)	1 (5%)	0 (0%)
CD4^+^ T-cell count (cells/mm^3^)			
***N***	*N* = 10	*N* = 16	*N* = 10
**Median ± SD**	859 ± 309	579 ± 302	775 ± 389
**(range, min to max)**	(525–1,567)	(337–1,500)	(358–1,454)
CD8^+^ T-cell count (cells/mm^3^)			
***N***	*N* = 10	*N* = 16	*N* = 10
**Median ± SD**	399 ± 461	822 ± 512	636 ± 403
**(range, min to max)**	(307–1,827)	(373–2,100)	(395–1,606)
Ratio of #CD4/#CD8 T-cell counts (cells/mm^3^)			
***N***	*N* = 10	*N* = 16	*N* = 10
**Median ± SD**	2.03 ± 0.64	0.71 **±** 0.37	1.04 **±** 0.56
**(range, min to max)**	(0.62–2.77)	(0.33–1.68)	(0.48–2.11)

### 3.2 Identification of lineage and immune cell subsets

PBMCs from 10 HIV-negative, 20 HIV+ cART-naïve, and 10 HIV+ pre-NHL (cART-naïve) participants were labeled with metal-tagged antibodies for mass CyTOF. The panel included lineage cell markers for B cells, CD3^+^ T cells (CD4^+^ and CD8^+^ T cells), and CD14^+^ monocytes; markers of B-cell activation and/or differentiation; and oncogenic markers (**Supplementary Table 2**). Before analysis, samples were normalized, and sample acquisition stability was confirmed (191Ir+ vs. time). Cells were gated using 191Ir+ vs. 193Ir+, doublets were discarded, and viable cells were gated as 194Pt- (**Supplementary Figure 1A**). CD14^-^CD11b^-^ cells were the base population utilized to determine the relative proportions of CD3^+^ T cells (CD14^-^CD11b^-^CD19^-^CD3^+^) and CD19^+^ B cells (CD14^-^CD11b^-^CD3^-^CD19^+^) (**Supplementary Figure 1B**). UMAP plots of singlets and viable cells showed clear resolution of major immune subsets CD19^+^ B cells, CD3^+^ T cells (CD4^+^CD8^-^ T cells; CD4^-^CD8^+^ T cells; CD4^+^CD8^+^ T cells; and CD3^+^ double-negative T cells), CD14^+^ monocytes, and other cell populations that were not identified by the antibody panel used in this study (**Supplementary Figures 1C, D**).

### 3.3 Characterization of CD19^+^ B cells

**Figure 1:**
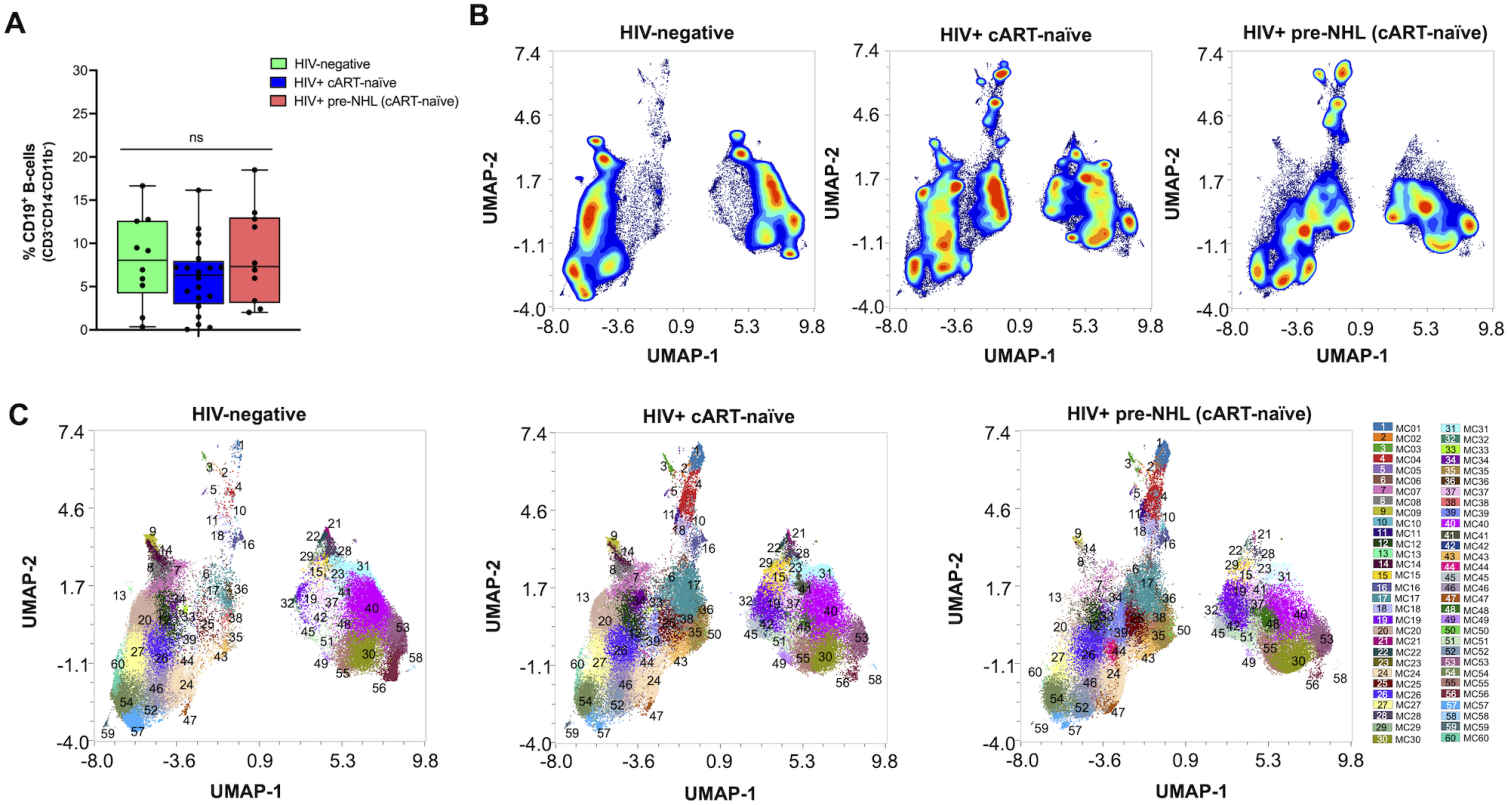
Unsupervised clustering of CD19^+^ B cells identifies 60 metaclusters in HIV-negative, HIV+ cART-naïve, and HIV+ pre-NHL (cART-naïve) samples. **(A)** Quantification and comparison in the percentage of CD19^+^ B cells (CD3^-^CD14^-^CD11b^-^) from total viable cells in PBMCs isolated from HIV-negative (*n* = 10), HIV^+^ cART-naïve (*n* = 20), and HIV^+^ pre-NHL (cART-naïve) (*n* = 10) (Mann–Whitney, unpaired non-parametric test, *p* > 0.05). **(B)** Contour plots of UMAPs for each group. UMAP plots were generated from an equal subsampling of 75,000 CD19^+^ B cells (CD3^-^CD14^-^CD11b^-^) for each group after manual gating (**Supplementary Figures 1A, B**). **(C)** Concatenated metacluster data [metacluster (MC) 1 to 60] shown as UMAP plots for each group; HIV-negative (*n* = 10), HIV+ cART-naïve (*n* = 20), and HIV+ pre-NHL (cART-naïve) (*n* = 10).

The purpose of this study was to conduct unsupervised clustering analysis of CD19^+^ B cells and describe phenotypically distinct B-cell populations in HIV+ cART-naïve and HIV+ pre-NHL (cART-naïve) that went on to develop NHL (median of 12 months before NHL diagnosis) (**Supplementary Table 2**). We first compared CD19^+^ B-cell proportions from viable cells in HIV-negative, HIV+ cART-naïve, and HIV+ pre-NHL (cART-naïve) samples. HIV+ pre-NHL (cART-naïve) trended to have elevated proportions of B cells compared to HIV+ cART-naïve (**Figure 1A**). For in-depth and high-dimensional analysis, we performed unsupervised clustering of CD19^+^ B cells from concatenated FCS data files for each group after mass cytometry: 10 HIV-negative (12 data files), 20 HIV+ cART-naïve samples (22 data files), and 10 HIV+ pre-NHL (cART-naïve) samples (11 data files). Prior to unsupervised clustering analysis, we confirmed that CD19^+^ B cells did not express CD3 and CD14 (non-B-cell exclusion markers) and CD11b (**Supplementary Figure 2A**). **Supplementary Figure 2B** provides histogram overlays of different markers of B-cell activation and/or differentiation, and oncogenic markers analyzed in this study. We performed unsupervised clustering on 75,000 CD19^+^ B cells per group using UMAP for dimension reduction and FlowSOM clustering algorithms in OMIQ. High-dimensional analysis of CD19^+^ B cells revealed 60 metaclusters in HIV-negative, HIV+ cART-naïve, and HIV+ pre-NHL (cART-naïve) samples (**Figures 1B, C**).

### 3.4 CD10^+^CD27^-^ B cells, CD20^+^CD27^-^ B cells, and B-cell populations with aberrant features (CD20^+^CD27^+^CXCR4^+^CD71^+^ B cells and CD20^+^CXCR4^+^cMYC^+^ B cells) are significantly elevated in HIV+ cART-naïve compared with HIV-negative samples

**Figure 2:**
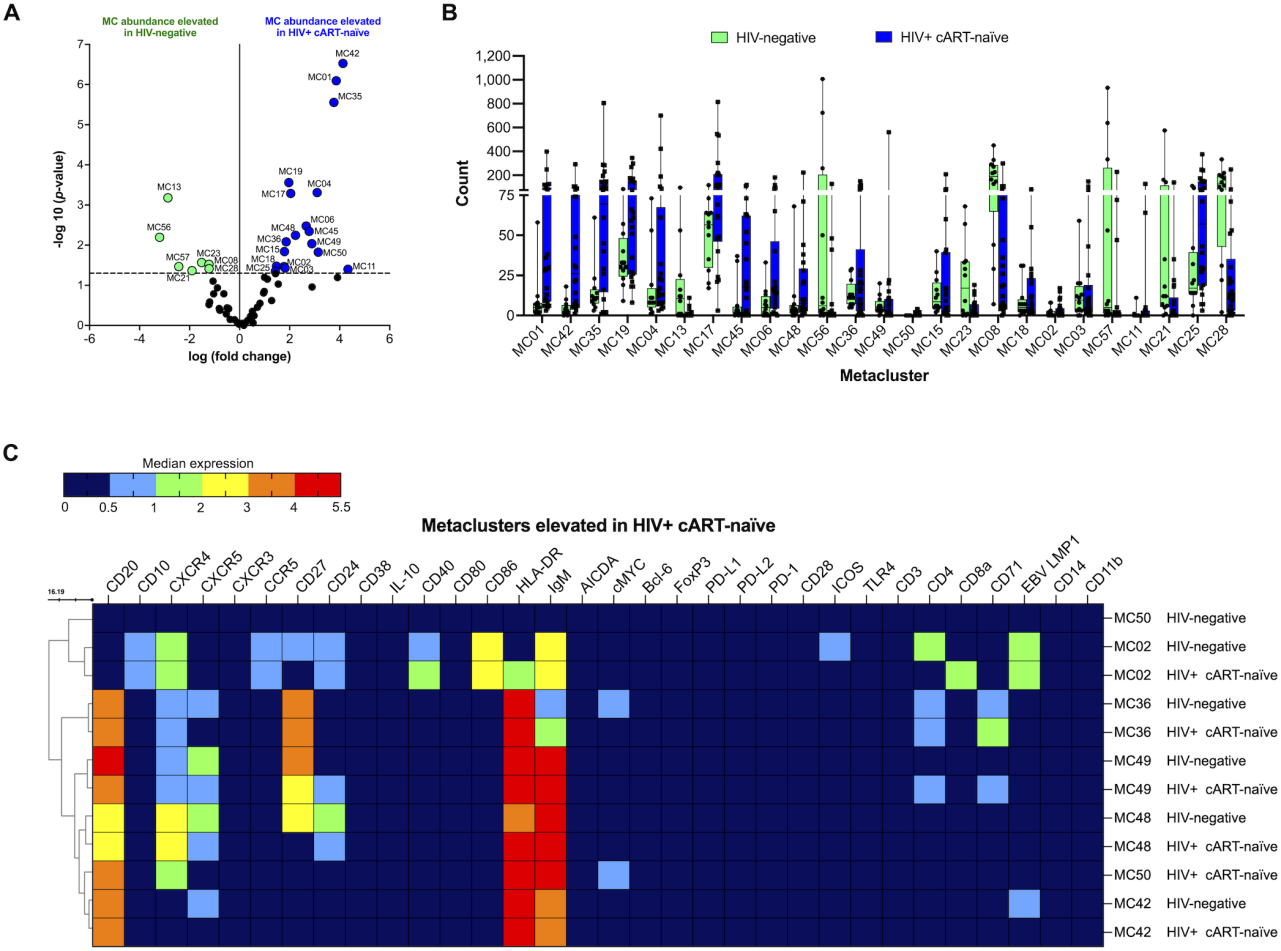
CD10^+^CD27^-^ B cells (MC02), CD20^+^CD27^+^CXCR4^+^CD71^+^ B cells (MC36 and MC49), CD20^+^CXCR4^+^cMYC^+^ B cells (MC50), and CD20^+^CD24^+^CXCR4^+^CXCR5^+^ B cells (MC48) are significantly elevated in HIV+ cART-naïve compared with HIV-negative. **(A)** Volcano plot showing log FC (log fold change) and adjusted *p*-values (*p* < 0.05) for statistically significant differences in B-cell metaclusters (MC) for HIV-negative and HIV+ cART-naïve samples. A total of 25 significant metaclusters are shown above the −log10 (*p*-value of 0.05) threshold (dotted line): 7 MCs for HIV-negative and 18 MCs for HIV+ cART-naïve. Data are for concatenated files of HIV-negative (*n* = 10) and HIV+ cART-naïve (*n* = 20). **(B)** Box plots showing cell counts within each significant metacluster shown in **(A)**. **(C)** Heatmap of median marker expression values in metaclusters identified in **(A)**. Concatenated data are summarized in a heatmap for selected metaclusters elevated in HIV+ cART-naïve compared to HIV-negative (MC02, MC36, MC49, MC48, MC50, and MC42).

We first compared CD19^+^ B cells in HIV-negative and HIV+ cART-naïve samples. After UMAP and FlowSOM, we visualized each metacluster population identified and conducted statistical analysis of the identified metaclusters. Overall, 25 metaclusters were significantly elevated at or above the threshold of −log10 (*p*-value < 0.05) or 1.30 (**Figure 2A**). Eighteen and seven metaclusters were significantly elevated in HIV+ cART-naïve and HIV-negative samples, respectively (**Figures 2A, B**). Of the 18 significantly elevated metaclusters in HIV+ cART-naïve samples, we identified four distinct metaclusters of CD20^+^CD27^-^ B cells (MC35, MC17, MC45, and MC48) and two distinct metaclusters of CD10^+^CD27^-^ B cells (MC02 and MC11) compared to HIV-negative samples, where all cell metaclusters expressed CD27 (**Figure 2C** and **Supplementary Table 3**). Given the CD27^-^ phenotype of these CD10^+^ B cells in HIV+ cART-naïve samples, we hypothesized that these cells represented a population of immature-like transitional B cells in the circulation of PLWH.

We further identified significantly elevated levels of two CD20^+^CXCR5^-^ B-cell populations in HIV+ cART-naïve compared to HIV-negative samples: CD20^+^CXCR5^-^IgM^+^HLA-DR^+^ B cells (MC42) and CD20^+^CD27^+^CXCR4^+^CXCR5^-^CD4^+^CD71^+^IgM^hi^HLA-DR^+^ B cells (MC36) (**Figure 2C** and **Supplementary Table 3**). The loss or impaired expression of CXCR5 on B cells during HIV infection has been reported by others (41). Of interest, we observed an elevated abundance of B cells expressing CD71 in HIV+ cART-naïve samples: CD20^+^CD27^+^CD24^+^CXCR4^+^CXCR5^+^CD4^+^CD71^+^IgM^+^HLA-DR^+^ B cells (MC49). The corresponding B cells in MC49 of HIV-negative samples did not express CD71 (**Figure 2C** and **Supplementary Table 3**). Elevated levels of mature B cells expressing CD71 may suggest a chronically activated state that can contribute to immune dysregulation in PLWH.

Moreover, a metacluster of CD20^+^CXCR4^+^cMYC^+^IgM^+^HLA-DR^+^ B cells was specifically present in HIV+ cART-naïve and absent in HIV-negative samples (MC50) (**Figure 2C**). The expression of cMYC reflects active proliferation and potential B-cell differentiation features. Other significantly elevated populations in HIV+ cART-naïve samples are summarized in **Supplementary Table 3**.

### 3.5 CD20^+^CD27^+^CD24^+^CXCR4^+^CXCR5^+^ B cells, CD20^+^CD27^+^CD10^+^CD24^+^CXCR4^+^ cMYC^+^ B cells, and populations of CD20^+^CD27^-^ B cells are significantly elevated in HIV+ pre-NHL (cART-naïve) compared to HIV+ cART-naïve samples

**Figure 3:**
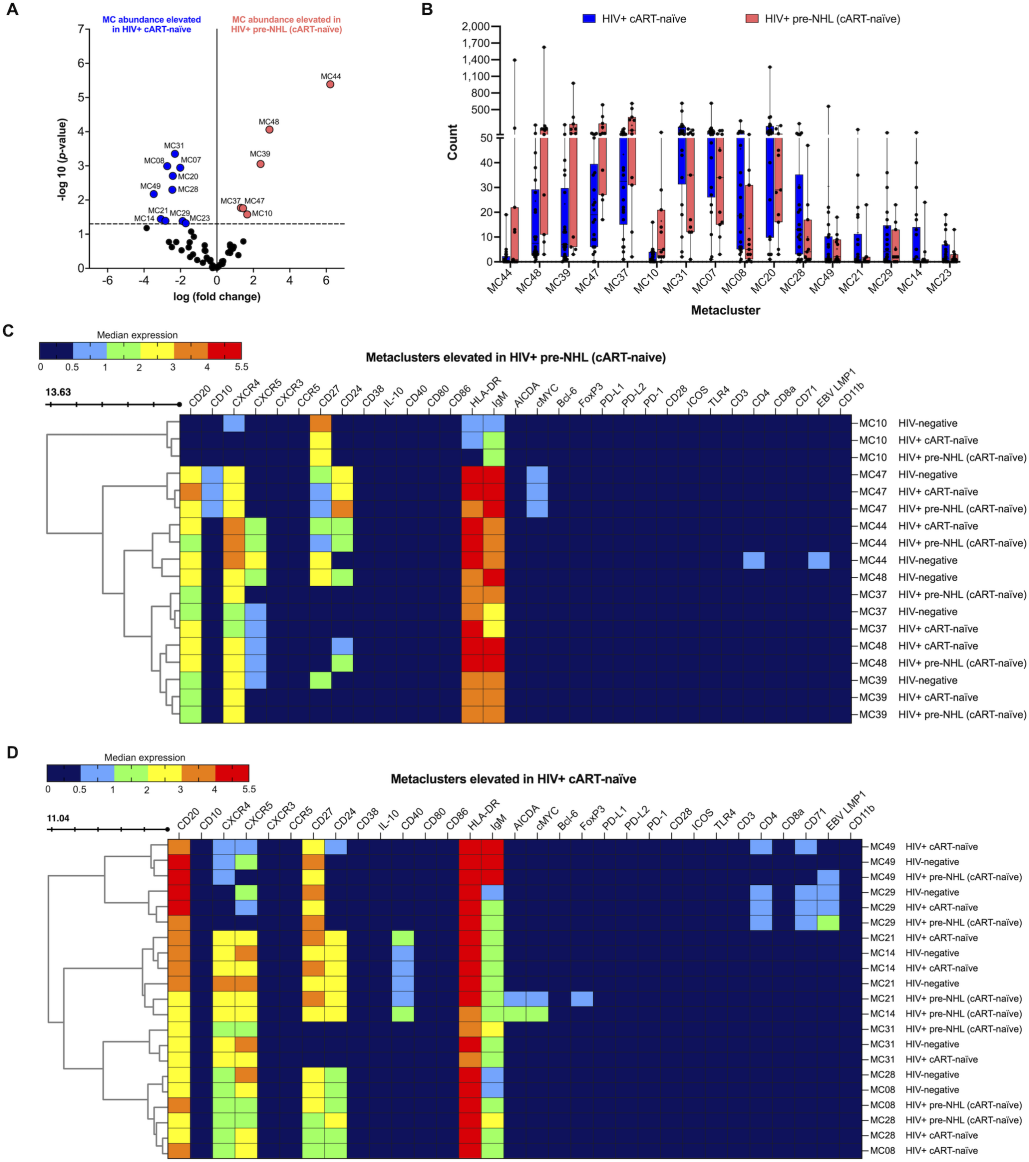
CD27^+^IgM^+^ B cells (MC10), CD20^+^CXCR4^+^ B cells (MC37 and MC39), CD20^+^CD27^+^CD24^+^CXCR4^+^cMYC^+^ B cells (MC47), and CD20^+^CD24^+^CXCR4^+^CXCR5^+^ B cells (MC44 and MC48) are significantly elevated in HIV+ pre-NHL (cART-naïve). **(A)** Volcano plot showing log FC (log fold change) and adjusted *p*-values (*p* < 0.05) for statistically significant differences in B-cell metaclusters for HIV+ cART-naïve and HIV+ pre-NHL (cART-naïve). A total of 16 significant metaclusters are shown above the −log10 (*p*-value of 0.05) threshold (dotted line): 10 MCs for HIV+ cART-naïve and 6 MCs for HIV+ pre-NHL (cART-naïve). Data are for concatenated files of HIV+ (*n* = 20) and HIV+ Pre-NHL (*n* = 10). **(B)** Box plots showing cell counts in each significant metacluster shown in **(A)**. **(C)** Concatenated data summarized in a heatmap with median marker expression values for metaclusters elevated in HIV+ pre-NHL (cART-naïve) compared to HIV+ cART-naïve (MC10, MC37, MC48, MC47, MC39, and MC44). **(D)** Heatmap of median marker expression values for the metaclusters elevated in HIV+ cART-naïve samples (MC29, MC49, MC14, MC21, MC08, MC28, and MC31).

We then compared B-cell metaclusters in HIV+ cART-naïve with HIV+ pre-NHL (cART-naïve) samples. Overall, 16 metaclusters were significantly elevated at or above the threshold of −log10 (*p*-value < 0.05) (**Figure 3A**). Ten and six metaclusters were significantly elevated in HIV+ cART-naïve and HIV+ pre-NHL (cART-naïve) samples, respectively (**Figures 3A, B**). A significantly elevated population of CD27^+^CXCR4^-^IgM^+^HLA-DR^+^ memory B cells was observed in HIV+ pre-NHL (cART-naïve) (MC10) compared to HIV+ cART-naïve samples (**Figure 3C** and **Supplementary Table 4**). Other significantly elevated populations of B cells in HIV+ pre-NHL (cART-naïve) were CD20^+^CXCR4^+^CXCR5^-^IgM^+^HLA-DR^+^ B cells (MC37); CD20^+^CD27^-^CXCR4^+^CXCR5^-^IgM^+^HLA-DR^+^ B cells (MC39); CD20^+^CD27^+^CD10^+^CD24^+^CXCR4^+^IgM^+^HLA-DR^+^ memory B cells expressing cMYC (MC47); CD20^+^CD27^+^CD24^+^CXCR4^+^CXCR5^+^ B cells (MC44); and CD20^+^CD27^-^CD24^+^CXCR4^+^CXCR5^+^ B cells (MC48) (**Figure 3C** and **Supplementary Table 4**).

### 3.6 CD20^+^CD27^+^CD71^+^ B cells and CD20^+^CD27^+^CD24^+^CD40^+^CXCR4^+^CXCR5^+^ B cells are significantly elevated in HIV+ cART-naïve compared to HIV+ pre-NHL (cART-naïve) samples

Further examination of select metaclusters significantly elevated in HIV+ cART-naïve samples showed an expansion of CD20^+^CD27^+^CXCR5^+^CD4^+^CD71^+^ B cells (MC29), CD20^+^CXCR4^+^CXCR5^+^ B cells (MC31), and distinct CD20^+^CD27^+^CD24^+^CXCR4^+^CXCR5^+^ B-cell populations (MC49, MC14, MC21, MC08, and MC28) (**Figure 3D** and **Supplementary Table 5**). Although significantly lower levels of CD20^+^CD27^+^CD24^+^CD40^+^CXCR4^+^CXCR5^+^ B cells were observed in MC14 of HIV+ pre-NHL (cART-naïve) samples, these cells had a potential pre-lymphoma phenotype as they expressed both cMYC and AICDA compared to HIV+ cART-naïve and HIV-negative samples (**Figure 3D** and **Supplementary Table 5**). The CD20^+^CD27^+^CD24^+^CD40^+^CXCR4^+^CXCR5^+^ B cells identified in MC21 of HIV+ pre-NHL (cART-naïve) samples also had a potential pre-lymphoma phenotype (cMYC^+^ and AICDA^+^), were FoxP3^+^, and had higher CD27 expression compared to its respective metacluster population in MC21 of HIV+ cART-naïve samples (*p* = 0.020) (**Figure 3D** and **Supplementary Table 5**). In addition, the cells in MC21 of HIV-negative samples did not express cMYC or AICDA.

We then evaluated whether the CD20^+^CD27^+^CD24^+^CD40^+^CXCR4^+^CXCR5^+^ B-cell populations expressing cMYC and AICDA in HIV+ pre-NHL (cART-naïve) samples (MC14 and MC21) had a potentially clonal origin by examination of Ig kappa and lambda light chain (LC). We looked at this on a case-by-case basis and found that the CD20^+^CD27^+^CD24^+^CD40^+^CXCR4^+^CXCR5^+^ B cells in MC14 of 1 of the 10 total HIV+ pre-NHL (cART-naïve) samples were specifically positive for Ig kappa LC and negative for Ig lambda LC (median expression value of Ig kappa LC = 3.40 vs. median expression value of Ig lambda LC = 0.24). The remaining HIV+ pre-NHL (cART-naïve) samples were either positive or negative for both Ig kappa and Ig lambda LC. Moreover, 4 of the 10 HIV+ pre-NHL (cART-naïve) samples had metaclusters of CD20^+^CD27^+^CD24^+^CD40^+^CXCR4^+^CXCR5^+^ B cells (MC21) that were positive for both Ig kappa and Ig lambda LC (median expression value of Ig kappa LC = 1.15 vs. median expression value of Ig lambda LC = 4.50; *p* = 0.029). The remaining six HIV+ pre-NHL (cART-naïve) samples were negative for both Ig kappa and Ig lambda LC.

### 3.7 CD20^+^CD86^+^ B cells, CD20^+^CD27^+^CD10^+^CD24^+^CXCR4^+^cMYC^+^ B cells, and populations of CD20^+^CD27^-^ and CD20^+^CXCR5^-^ B cells are elevated in HIV+ pre-NHL (cART-naïve) compared to HIV-negative samples

**Figure 4:**
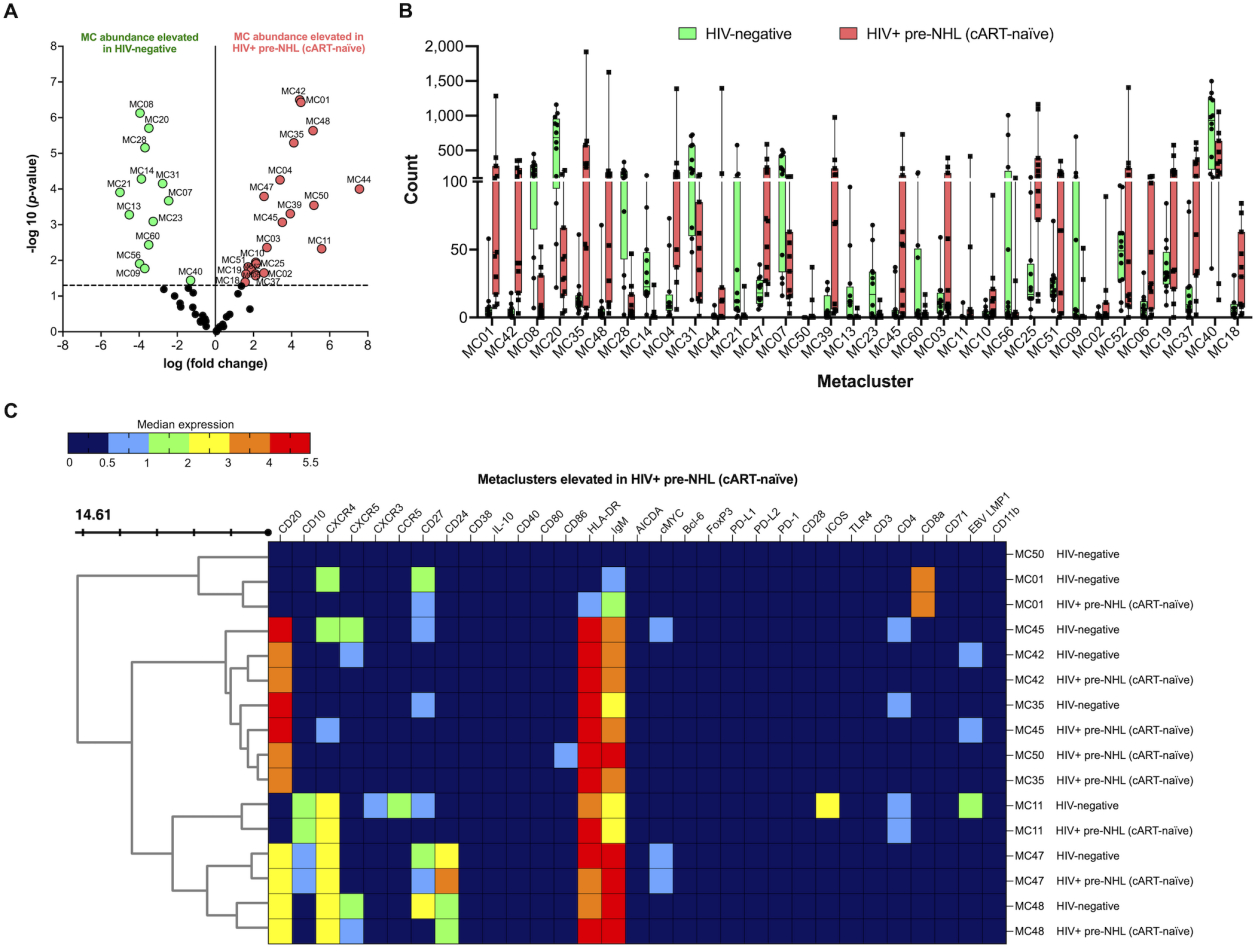
CD10^+^CXCR4^+^ B cells (MC11, MC47), CD20^+^CD27^-^ B cells (MC35), CD20^+^CXCR4^+^ B cells (MC45), CD20^+^CD24^+^CXCR4^+^CXCR5^+^ B cells (MC48), and CD20^+^CD86^+^ B cells (MC50) are significantly elevated in HIV+ pre-NHL (cART-naïve) compared with HIV-negative. **(A)** Volcano plot showing log FC (log fold change) and adjusted *p*-values (*p* < 0.05) for statistically significant differences in B-cell metaclusters for HIV-negative and HIV+ pre-NHL (cART-naïve). A total of 34 significant metaclusters are shown above the −log10 (*p*-value of 0.05) threshold (dotted line):13 MCs for HIV-negative and 21 MCs for HIV+ pre-NHL (cART-naïve). Data are for concatenated files of HIV-negative (*n* = 10) and HIV+ pre-NHL (cART-naïve) (*n* = 10). **(B)** Box plots showing cell counts for each significant metacluster shown in **(A)**. **(C)** Concatenated data summarized in a heatmap with median marker expression values in select metaclusters elevated in HIV+ pre-NHL (cART-naïve) (MC11, MC35, MC42, MC47, MC48, MC50, MC01, and MC45).

When we compared the unsupervised clustering results of CD19^+^ B cells from HIV-negative and HIV+ pre-NHL (cART-naïve) samples, we found that 21 metaclusters were significantly elevated in HIV+ pre-NHL (cART-naïve) samples (**Figures 4A, B**). We identified significantly elevated levels of CD20^+^CD27^+^CD10^+^CD24^+^CXCR4^+^cMYC^+^ B cells (MC47) and populations of CD20^+^CD27^-^ (MC48, MC35, MC39, and MC45) and CD20^+^CXCR5^-^ B cells (MC42, MC06, and MC37) (**Figure 4C** and **Supplementary Table 6**). We also identified CD10^+^CXCR4^+^ B cells lacking CD27, CXCR3, CCR5, and ICOS expression in HIV+ pre-NHL (cART-naïve) compared to HIV-negative samples (MC11) (**Figure 4C** and **Supplementary Table 6**). Other significantly elevated populations included CD20^+^CD27^-^CD24^+^CXCR4^+^CXCR5^+^IgM^+^ B cells (MC48) with elevated HLA-DR expression compared to HIV-negative samples (*p* = 0.001), a metacluster of CD27^+^CXCR4^-^CD8^+^HLA-DR^+^ B cells (MC01) with elevated IgM expression (*p* = 0.013), and a metacluster of CD20^+^CD86^+^IgM^+^HLA-DR^+^ B cells that was specifically absent in HIV-negative samples (MC50) (**Figure 4C** and **Supplementary Table 6**). Other significantly elevated metaclusters in HIV+ pre-NHL (cART-naïve) samples and their phenotypes are summarized in **Supplementary Table 6**.

### 3.8 CD19^-^CD20^+^ B cells show an activated memory phenotype in HIV+ cART-naïve and HIV+ pre-NHL (cART-naïve) samples

Maturing B cells show lower levels of CD19 expression and higher levels of CD20 (42). To examine potential differences in mature B-cell subsets of HIV+ cART-naïve and HIV+ pre-NHL (cART-naïve), we conducted unsupervised clustering of CD19^-^CD20^+^ B cells (**Supplementary Figures 3A, B**). We did not observe significant differences in CD19^-^CD20^+^ B-cell levels between HIV+ cART-naïve and HIV+ pre-NHL (cART-naïve) samples (**Supplementary Figure 3C**). Unsupervised clustering identified two significantly elevated metaclusters in HIV+ pre-NHL (cART-naïve) and four significantly elevated metaclusters in HIV+ (cART-naïve) samples (**Supplementary Figures 4A, B**). The metaclusters elevated in HIV+ pre-NHL (cART-naïve) samples included CD20^+^CXCR4^+^CD24^-^IgM^+^HLA-DR^+^ (MC08) and CD20^+^CXCR4^+^CD27^-^CD24^+^IgM^+^HLA-DR^+^ (MC01) B-cell populations (**Supplementary Figure 4C**). When we examined the metaclusters elevated in HIV+ cART-naïve samples, we identified a metacluster of CD20^+^CXCR4^+^ B cells that was PD-L1^+^ and Bcl-6^+^ in both HIV+ cART-naïve and HIV+ pre-NHL (cART-naïve) samples (MC12) (**Supplementary Figure 4D**). However, this specific B-cell population was uniquely cMYC^+^ in HIV+ pre-NHL (cART-naïve). In addition, significantly elevated levels of CD20^high^CXCR4^+^Bcl-6^+^ B cells with low-level CD4 and CD8 expression were identified in HIV+ cART-naïve samples (MC14) [CD20 marker expression: HIV+ cART-naïve vs. HIV+ pre-NHL (cART-naïve), *p* = 0.040]. The CD20^+^CXCR4^+^PD-L1^+^ B cells in MC12 and MC14 of HIV-negative samples displayed higher Bcl-6 expression compared to HIV+ cART-naïve (*p* = 0.009, MC12 and *p* = 0.003, MC14) and HIV+ pre-NHL (cART-naïve) (*p* = 0.011, MC12 and *p* = 0.001, MC14).

This analysis also identified significantly elevated levels of CD20^+^CXCR4^+^CXCR5^+^IgM^+^HLA-DR^+^ B cells (MC15) and CD20^+^CXCR4^+^CD4^+^IgM^+^HLA-DR^+^ B cells (MC16) in HIV+ cART-naïve samples. Interestingly, the CD20^+^CXCR4^+^CD4^+^ B cells identified in MC16 of HIV+ pre-NHL (cART-naïve) were CCR5^+^ compared to HIV+ cART-naïve and HIV-negative samples (**Supplementary Figure 4D**). The CD20^+^CXCR4^+^CD4^+^ B cells in MC16 of HIV-negative displayed elevated Bcl-6 expression compared to HIV+ cART-naïve (*p* < 0.0001) and HIV+ pre-NHL (cART-naïve) samples (*p* = 0.005).

### 3.9 Examination of CD20^+^CXCR4^hi^ B cells reveals potential pre-lymphoma phenotypes in HIV+ pre-NHL (cART-naïve) samples

**Figure 5:**
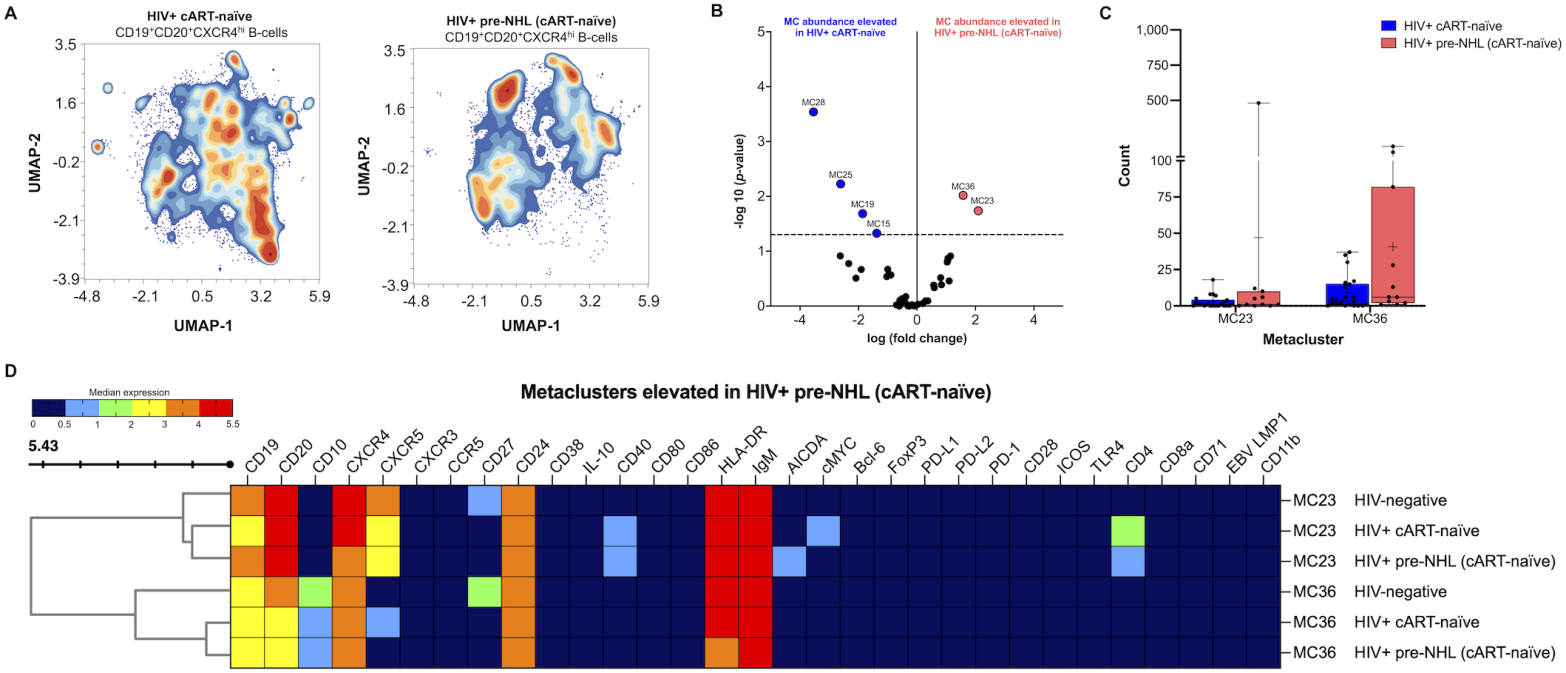
Elevated levels of CD20^+^CXCR4^hi^ B cells expressing CD24 and/or AICDA are observed in HIV+ pre-NHL (cART-naïve). **(A)** Unsupervised clustering of CD19^+^CD20^+^CXCR4^hi^ B cells provide 38 metaclusters in HIV+ cART-naïve and HIV+ pre-NHL (cART-naïve), and contour plots are shown. UMAP plots were generated from an equal subsampling of 12,500 CD19^+^CD20^+^CXCR4^hi^ B cells for each group. UMAP plots are for concatenated files of HIV+ cART-naïve (*n* = 20) and HIV+ pre-NHL (cART-naïve) (*n* = 10). **(B)** Volcano plot showing significantly elevated metaclusters in HIV+ cART-naïve (MC28, MC25, MC19, and MC15) and HIV+ pre-NHL (cART-naïve) (MC23 and MC36). Significant metaclusters are shown above the −log10 (*p*-value of 0.05) threshold (dotted line). **(C)** Box plots showing cell counts for the two significantly elevated metaclusters in HIV+ pre-NHL (cART-naïve) (MC23 and MC36) compared to those metaclusters in HIV+ cART-naïve samples. **(D)** Clustered heatmap with median marker expression values of metaclusters 36 and 23.

Significantly elevated metaclusters identified in the unsupervised clustering analysis of CD19^+^ B cells from HIV+ pre-NHL (cART-naïve) had CD20^+^CXCR4^+^ phenotypes (**Figure 3C**). Thus, we performed unsupervised clustering of CD20^+^CXCR4^hi^ B cells in both HIV+ cART-naïve and HIV+ pre-NHL (cART-naïve) samples (**Figure 5A**). The gating strategy of CD20^+^CXCR4^hi^ B cells is summarized in **Supplementary Figure 5**. We did not observe significant differences in the levels of CD20^+^CXCR4^+^ or CD20^+^CXCR4^hi^ B cells between HIV+ cART-naïve and HIV+ pre-NHL (cART-naïve) samples (**Supplementary Figures 5C, D**). However, after unsupervised clustering, we identified two significantly elevated CD20^+^CXCR4^hi^ B-cell populations in HIV+ pre-NHL (cART-naïve) compared to HIV+ cART-naïve samples (MC23 and MC36) (**Figures 5B, C**). The CD20^+^CXCR4^hi^CD24^+^CXCR5^+^CD40^+^CD4^+^ B-cell population in MC23 of HIV+ pre-NHL (cART-naïve) samples displayed a mature and activated phenotype (IgM^+^HLADR^+^) with a potential pre-lymphoma feature as it expressed AICDA compared to MC23 of HIV+ cART-naïve samples. This population also displayed a CD27^-^ phenotype compared to MC23 of HIV-negative samples (**Supplementary Table 7**). The CD20^+^CXCR4^hi^CD27^-^CD24^+^CXCR5^+^CD40^+^CD4^hi^ B cells in MC23 of HIV+ cART-naïve samples specifically expressed cMYC compared to HIV-negative and HIV+ pre-NHL (cART-naïve) samples. Moreover, CD20^+^CXCR4^hi^CD10^+^CD24^+^IgM^+^HLA-DR^hi^ B cells in MC36 of HIV+ pre-NHL (cART-naïve) samples displayed CD27^-^ and CXCR5^-^ phenotypes compared to HIV-negative samples (**Figure 5C** and **Supplementary Table 7**). The CD20^+^CXCR4^hi^CD10^+^CD27^-^CD24^+^IgM^+^HLA-DR^+^ B cells in MC36 of HIV+ cART-naïve samples expressed CXCR5 compared to HIV+ pre-NHL (cART-naïve) samples. Loss of CD27 and CXCR5 expression in these B-cell populations indicates potential immune dysfunction and may be more prone to malignant transformation in untreated HIV+ individuals.

We also identified four metaclusters significantly elevated in HIV+ cART-naïve compared to HIV+ pre-NHL (cART-naïve) samples. Of interest, we identified a CD20^+^CXCR4^hi^ metacluster expressing CD40 and CD4 compared to HIV-negative samples (MC19) and a significant expansion of CD20^+^CXCR4^hi^CD27^+^CD24^+^CXCR5^+^CD4^+^cMYC^+^IgM^hi^ HLA-DR^+^ B cells (MC15) compared to HIV-negative and HIV+ pre-NHL (cART-naïve) samples (**Supplementary Table 7**).

### 3.10 CD19^+^CD24^hi^CD38^hi^ Bregs are significantly elevated in HIV+ pre-NHL (cART-naïve) samples and have malignant, pre-lymphoma phenotypes

**Figure 6:**
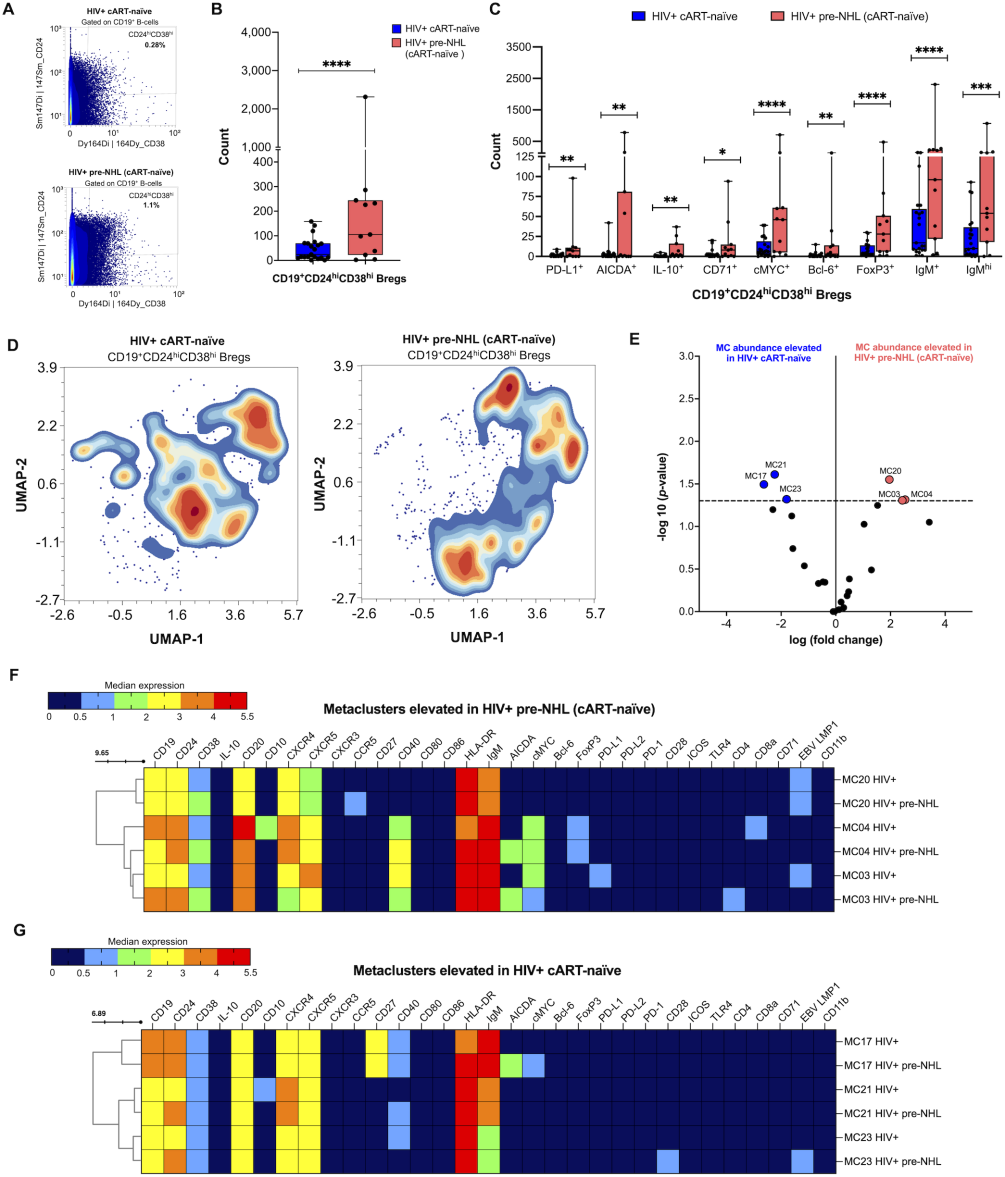
Bregs are significantly elevated in HIV+ pre-NHL (cART-naïve) and have unique pre-lymphoma phenotypes. **(A)** CD24^hi^CD38^hi^ cells were manually gated from CD19^+^ B cells (CD3^-^CD14^-^CD11b^-^) from PBMCS of HIV+ cART-naïve (*n* = 20) and HIV+ pre-NHL (cART-naïve) (*n* = 10) samples. Shown is a representative plot of Bregs for each group with percent values. **(B)** Counts of manually gated Bregs in HIV+ cART-naïve (*n* = 20) and HIV+ pre-NHL (cART-naïve) (*n* = 10) (Mann–Whitney, unpaired non-parametric test, ***p < 0.0005). **(C)** Counts of PD-L1+, AICDA+, IL-10+, CD71+, cMYC+, Bcl-6+, FoxP3 +, IgM+, or IgMhi Bregs in HIV+ cART-naïve and HIV+ pre-NHL (cART-naïve) samples (Mann–Whitney, unpaired non-parametric test, *p < 0.05; **p<0.005; ***p < 0.0005; ****p<0.00005). **(D)** Unsupervised clustering of Bregs provided 25 metaclusters in HIV+ cART-naïve and HIV+ pre-NHL (cART-naïve). UMAPs were generated from an equal subsampling of 5,000 Bregs for each group. Contour plots are shown. **(E)** Volcano plot with significantly elevated metaclusters in HIV+ cART-naïve (MC17, MC21, and MC23) and HIV+ pre-NHL (cART-naïve) (MC20, MC04, and MC03) [shown above the −log10 (*p*-value of 0.05) threshold, dotted line]. **(F)** Heatmap of median marker expression values of significantly elevated metaclusters in HIV+ pre-NHL (cART-naïve) (MC20, MC04, and MC03). **(G)** Heatmap of median marker expression values of significantly elevated metaclusters in HIV+ cART-naïve samples (MC17, MC21, and MC23).

Our previous work showed that Bregs are elevated in PLWH and 1 to 4 years prior to an AIDS-NHL diagnosis compared to HIV+ controls that did not go on to develop lymphoma (33). Bregs exert immunoregulatory functions and may express molecules involved in both mounting and suppressing immune responses, including immune regulatory ligands such as PD-L1 (33), and potentially express aberrant or oncogenic markers. Therefore, we conducted in-depth phenotyping of Bregs in HIV+ cART-naïve and HIV+ pre-NHL (cART-naïve) samples. First, we manually gated Bregs as CD24^hi^ and CD38^hi^ expression (**Figure 6A**). We found significantly elevated levels of CD19^+^CD24^hi^CD38^hi^ Bregs in HIV+ pre-NHL (cART-naïve) compared to HIV+ cART-naïve samples (**Figure 6B**). We then manually gated for PD-L1 and pre-lymphoma markers, such as AICDA and cMYC (**Supplementary Figure 6**). We found that HIV+ pre-NHL (cART-naïve) samples had significantly elevated levels of PD-L1^+^, AICDA^+^, IL-10^+^, CD71^+^, cMYC^+^, Bcl-6^+^, FoxP3^+^, and IgM^+^/IgM^hi^ Bregs compared to HIV+ cART-naïve samples (**Figure 6C**). After performing unsupervised clustering of 5,000 Bregs for each group (**Figure 6D**), we identified 25 metaclusters in both HIV+ cART-naïve and HIV+ pre-NHL (cART-naïve) samples. Of the 25 metaclusters, 3 were significantly elevated in both HIV+ pre-NHL (cART-naïve) (MC20, MC04, and MC3) and HIV+ cART-naïve (MC21, MC17, and MC23) samples (**Figure 6E**). Significantly elevated levels of Bregs expressing CD20, CXCR4, CXCR5, and CCR5 were observed in MC20 of HIV+ pre-NHL (cART-naïve) samples (**Figure 6F**). These Bregs also expressed IgM and HLA-DR and were EBV LMP1+. The second elevated Breg population in HIV+ pre-NHL (cART-naïve) samples expressed CD20, CXCR5, CD40, and FoxP3; were cMYC^+^; and were uniquely AICDA^+^ compared to HIV+ cART-naïve samples (MC04) (**Figure 6F**). The third elevated Breg population in HIV+ pre-NHL (cART-naïve) samples also expressed CD20, CXCR4, CXCR5, CD40; were cMYC^+^; and were uniquely AICDA^+^ and CD4^+^ compared to HIV+ cART-naïve samples (MC03) (**Figure 6F**).

Closer examination of metaclusters significantly elevated in HIV+ cART-naïve samples showed elevated levels of Bregs expressing CD20, CXCR4, CXCR5, and CD40 (MC17 and MC23) and Bregs expressing CD20, CD10, CXCR4, and CXCR5 (MC21) (**Figure 6G**). All Breg metaclusters had an activated memory phenotype as they were IgM^+^ and HLA-DR^+^. Although Bregs present in MC17 of HIV+ pre-NHL (cART-naïve) were not significantly elevated relative to HIV+ cART-naïve samples, they had a potential pre-lymphoma phenotype as they were uniquely cMYC^+^ and had significant AICDA positivity (*p* = 0.031) (**Figure 6G**).

These findings suggest that AICDA^+^ Breg cells may play an important role in the expansion of pre-malignant B cells in HIV+ and cART-naïve individuals that go on to develop NHL.

## 4 Discussion

Phenotypically aberrant B cells and B-cell populations not commonly present in healthy individuals, such as immature transitional B cells, exhausted B cells, and activated mature B cells, have been observed in PLWH (43). HIV infection induces the expression of B-cell activation markers, polyclonal B-cell activation, and the differentiation of B cells, which increase the incidence of B-cell malignancies (16). In this study, we sought to elucidate B-cell populations with pre-lymphoma or aberrant phenotypes in the circulation of HIV+ cART-naïve individuals and HIV+ cART-naïve individuals that go on to develop NHL, and prior to their diagnosis. Archival, viably frozen PBMCs were obtained from the Los Angeles site of the MWCCS cohort. We used mass cytometry to conduct high-dimensional analysis of single cells to measure quantitative and phenotypic differences in B-cell metaclusters identified for each cohort group. This approach allowed for the simultaneous examination of markers important for B-cell activation and/or differentiation, and the expression of oncogenic markers, such as cMYC, Bcl-6, and AICDA. We and others have shown that HIV infection induces B-cell hyperactivation and chronic production of B-cell stimulatory cytokines, which are particularly elevated in PLWH that go on to develop AIDS-NHL (16, 44), and years prior to an AIDS-NHL diagnosis (14, 23).

Here, we performed unsupervised analysis of high-dimensional mass cytometry data for CD19^+^ B cells. We first found that HIV+ pre-NHL (cART-naïve) individuals trended to elevated proportions of CD19^+^ B cells compared with HIV+ cART-naïve individuals. When we performed unsupervised clustering of CD19^+^ B cells and compared HIV+ cART-naïve with HIV-negative samples, we found elevated levels of CD10^+^CD27^-^ B cells in the circulation of HIV+ cART-naïve individuals, a phenotype associated to immature-like transitional B cells (43). In contrast, this specific metacluster consisted of CD10^+^CD27^+^ B cells in HIV-negative samples. The identification of a second CD10^+^CD27^-^ B-cell population elevated in HIV+ pre-NHL (cART-naïve) samples (MC11) (**Figure 4C**) suggests that immature transitional B cells play a role in the early stages of lymphomagenesis. CD10 is a surface metalloendopeptidase expressed on different cell types, including immature B cells and GC B cells, and is important in the early stages of B-cell differentiation, for example, during the first stage of immunoglobulin heavy chain rearrangement in pre-B cells (45). However, deregulated expression of CD10 plays a role in the early B-cell developmental stages of acute lymphoblastic leukemia and other hematologic malignancies of B-cell origin, such as follicular lymphoma, BL, and DLBCL (46, 47).

We previously showed that the proportion of circulating B cells that expressed CD27 was significantly reduced in HIV infection and serum levels of soluble CD27 (sCD27) were significantly elevated in HIV+ subjects who went on to develop AIDS-NHL compared to HIV+ subjects who did not develop lymphoma (48). Changes or deficiencies in B-cell subsets are known to occur in PLWH, including the loss of memory B cells and as measured by the expression of CD27 (49).

HIV infection causes disruption and structural changes in lymphoid tissue, such as histoarchitectural deterioration and fibrosis, altered intercellular communication between lymphocytes and stromal cells, and alterations in germinal center structure (50). The germinal center is a specialized microenvironment in secondary lymphoid tissues that produces long-lived antibody secreting plasma cells and memory B cells. Disruption of these structures may lead to the circulation of GC B cells or aberrant B cells in PLWH. To this point, we found elevated levels of activated memory B-cell subsets with potential aberrant or malignant phenotypes: CD20^+^CD27^+^CXCR4^+^CXCR5^-^CD4^+^CD71^+^IgM^hi^HLA-DR^+^ B cells (MC36) and, specifically, CD20^+^CXCR4^+^cMYC^+^IgM^+^HLA-DR^+^ B cells (MC50) in HIV+ cART-naïve samples compared to HIV-negative samples (**Figure 2C**). We also found significantly elevated levels of CD20^+^CD27^+^CXCR4^+^CXCR5^+^CD4^+^ B cells in MC49 of HIV+ cART-naïve samples that uniquely expressed CD24 and CD71 compared to HIV-negative samples. This B-cell population seen in the circulation of HIV+ cART-naïve samples is an aberrant memory B-cell population that is not observed in HIV-negative samples. CD24 is expressed in differentiating and maturing B cells (i.e., late pro-B cell stage), and its expression fluctuates throughout the lifespan of mature B cells such that expression is lost when B cells differentiate into antibody-producing cells (51). Moreover, the transferrin receptor CD71 is a mediator of iron uptake in different cell types, and in the context of B cells, it has been defined as an important activation marker. We have previously shown that CD71-expressing B cells are significantly elevated in PLWH before they develop AIDS-NHL (18).

We found that CD20^+^CD27^+^CD24^+^CXCR4^+^CXCR5^+^ B cells, CD20^+^CD27^+^CD10^+^CD24^+^ CXCR4^+^cMYC^+^ B cells, and populations of CD20^+^CD27^-^ B cells were significantly elevated in HIV+ pre-NHL (cART-naïve) compared to HIV+ cART-naïve samples (**Figure 3C** and **Supplementary Table 4**). A specific population of CD20^+^CXCR4^+^CXCR5^-^ B cells was also elevated in HIV+ pre-NHL (cART-naïve) (MC37) when compared to HIV+ cART-naïve samples.

CD20 is involved in B-cell activation and differentiation, and its expression is lost in terminally differentiated plasmablasts, but it is also expressed in malignant B cells; thus, it is a widely used diagnostic marker and therapeutic target for B-cell lymphomas (42). HIV uses its main receptor, CD4, and CXCR4 and CCR5 as coreceptors for entry into target CD4^+^ T cells (52). In the context of B-cell biology and disease, CXCR4 has been implicated in the migration and trafficking of malignant B cells in hematological malignancies, such as non-Hodgkin lymphoma and chronic lymphocytic leukemia (28, 53). The high expression of CXCR4 has been significantly associated with poor outcome of R-CHOP-treated DLBCL patients, and it was found to be an independent factor predicting poorer progression-free survival in germinal-center B-cell-like DLBCL (54), and shorter overall survival and progression-free survival in DLBCL patients (55).

When we examined the metaclusters significantly elevated in HIV+ cART-naïve samples, we specifically identified CD20^+^CXCR5^+^ B-cell populations (MC29 and MC49) that were negative for CXCR5 in HIV+ pre-NHL (cART-naïve). Decreased surface expression of CXCR5 by B cells has been reported for HIV-infected individuals with high-level viremia and thought to be due to ligand-induced internalization of CXCR5 and elevated levels of its ligand CXCL13 in serum of PLWH (41). Interestingly, CXCL13 is elevated in the circulation of HIV+ pre-NHL (cART-naïve) individuals perhaps, contributing to the lower expression of CXCR5 in these individuals in MC29 and MC49 (37). Although we do not have complete information on the viral load of these cohort samples, we can speculate that these specific CD20^+^CXCR5^-^ B-cell populations may have internalized CXCR5 during chronic infection. Defining associations between these B-cell populations and matched serum levels of CXCL13 would be important as we and others have shown that the levels of CXCL13 are significantly elevated in PLWH prior to an AIDS-NHL diagnosis (36, 37, 41, 56–58) and before and after the initiation of treatment of AIDS-NHL patients (59).

Moreover, we specifically found elevated levels of CD20^+^CD27^+^CD24^+^CXCR4^+^CXCR5^+^ B cells in HIV+ cART-naïve samples (MC28) (**Figure 3D** and **Supplementary Table 5**). CD20^+^CD27^+^CXCR4^+^CXCR5^+^ B cells in MC28 of HIV+ pre-NHL (cART-naïve) samples had elevated CD24 expression. This phenotype resembles a population of CD27^+^CD24^hi^ B cells previously identified in patients with autoimmune disease and defined as potential memory B cells (34). CD24 regulates cell migration, invasion, and cell proliferation, and has been shown to be highly expressed on different types of cancer cells of solid tumors (60).

We further identified two distinct CD20^+^CD27^+^CD24^+^CD40^+^CXCR4^+^CXCR5^+^ B-cell populations that were significantly elevated in HIV+ cART-naïve samples (MC14 and MC21), but that specifically expressed AICDA and cMYC in HIV+ pre-NHL (cART-naïve) samples (**Figure 3D** and **Supplementary Table 5**). In addition, we found that the CD20^+^CXCR4^+^CXCR5^+^ B-cell population in MC14 of 1 of the 10 HIV+ pre-NHL (cART-naïve) samples were potentially of clonal origin as they specifically showed Ig kappa LC positivity, and this clone had potential pre-lymphoma characteristics as it expressed both cMYC and AICDA. These findings suggest that pools of potentially clonal and pre-malignant B-cell subsets with oncogenic features are present in the circulation of PLWH before they are diagnosed with AIDS-NHL. Our results are also in accordance with our previous work demonstrating that AIDCA expression is induced in PBMCs of HIV+ cART-naïve individuals that go on to develop AIDS-NHL (18, 23).

When we conducted unsupervised clustering analysis of CD19^-^CD20^+^ B cells, we identified a metacluster of CD19^-^CD20^+^CXCR4^+^Bcl-6^+^PD-L1^+^ cells that were significantly elevated in HIV+ cART-naïve samples, but these cells uniquely expressed cMYC in HIV+ pre-NHL (cART-naïve) samples (**Supplementary Figure 4**). BCL-6 functions as a master regulator of GC responses and has been further characterized as a frequently translocated locus in DLBCLs (61). PD-L1 is an important immune-checkpoint molecule that has been shown to be elevated in B cells of HIV+ individuals that go on to develop AIDS-NHL (33), and the PD-L1 and PD-1 axis is potentially responsible for immune evasion in HIV-associated B-cell lymphomas (62). Elevated levels of CD20^+^CXCR4^+^ B cells expressing PD-L1 and Bcl-6 may signify a functionally distinct subset in HIV+ individuals potentially involved in immune regulation and antibody production, and that may contribute to disease progression and/or risk of malignancy in PLWH.

CXCR4-SDF-1 signaling in B cells leads to transcriptional activation and CD20 expression (42). There is also evidence that polymorphisms in the SDF-1 gene (G-to-A transition at position 801; SDF-3’A) increase NHL risk (63). When we specifically evaluated CD20^+^ and CXCR4 high-expressing B cells by unsupervised clustering, we identified elevated levels of CD20^+^CXCR4^hi^AICDA^+^ B cells in HIV+ pre-NHL (cART-naïve) compared to HIV+ samples (MC23) (**Figure 5D**). Increased expression of CXCR4 may negatively impact anti-CD20 response or R-CHOP chemotherapy of DLBCL patients (64). Thus, further characterization of CD20^+^CXCR4^hi^AICDA^+^ B cells in HIV+ pre-NHL may provide additional insight into events leading to B-cell transformation, CSR, and somatic mutations leading to carcinogenesis and/or contributing to the early stages of lymphomagenesis.

We and others have shown that CD19^+^CD24^hi^CD38^hi^ Bregs are elevated in HIV+ individuals and prior to an AIDS-NHL diagnosis (33, 65). In this study, we performed an unsupervised clustering analysis of Bregs for our cohort of PLWH to identify potential malignant or oncogenic features and to provide additional insight on this unique subset during the pre-lymphoma stage. We first observed elevated counts of CD19^+^CD24^hi^CD38^hi^ Bregs in the circulation of HIV+ pre-NHL compared to HIV+ samples (**Figure 6B**). Here, we found elevated levels of CD19^+^CD24^hi^CD38^hi^ Bregs that were IL-10^+^ in HIV+ pre-NHL (cART-naïve) samples. This finding suggests that IL-10-producing cells in the CD19^+^CD24^hi^CD38^hi^ Breg compartment of pre-NHL may play an important role in B-cell mediated regulatory mechanisms in lymphomagenesis.

We also found that CD19^+^CD24^hi^CD38^hi^ Bregs express phenotypic aberrant markers in both HIV+ cART-naïve and HIV+ pre-NHL (cART-naïve) samples, such as PD-L1, cMYC, AICDA, CD71, or Bcl-6, but significantly higher levels of these Breg cell subsets were found in HIV+ pre-NHL (cART-naïve) samples (**Figure 6C**). Unsupervised clustering revealed a significantly elevated metacluster of Bregs that were specifically cMYC^+^ AICDA^+^ in HIV+ pre-NHL (cART-naïve) compared to HIV+ cART-naïve samples (MC03 and MC04) (**Figure 6F**). When examining significantly elevated Breg metaclusters in HIV+ cART-naïve samples, we found that the cells in MC17 of HIV+ pre-NHL (cART-naïve) samples specifically expressed cMYC and AICDA (**Figure 6G**).

Our results indicate a potential role for Bregs in the initiation or in the pre-lymphoma stage of PLWH. Bregs can promote tumor growth by suppressing immunity through the inactivation of effector T and NK cells in the tumor environment (66). The elevated levels of Bregs expressing PD-L1 in HIV+ pre-NHL (cART-naïve) may play a role in lymphomagenesis by impairing/inhibiting T-cell function, including cytotoxic T cells (CTLs). These results are corroborated by previous findings where PLWH have elevated levels of PD-L1 expressing Bregs prior to an AIDS-NHL diagnosis (33). Pre-tumor Bregs expressing PD-L1 may contribute to early events in lymphomagenesis, such as the growth and expansion of malignant clones.

In future work, it will be important to describe IL-10-producing B cells, such as CD138^+^ Bregs (IL-10^+^CD138^+^) or IL-10-producing plasma cells, which have been ascribed to exert regulatory functions in autoimmune inflammation (67) and other autoimmune diseases (68). In addition, IL-10 signaling has been shown to promote lymphoma growth *in vivo* and tumor-derived IL-10 promotes immune escape in DLBCL (69). Moreover, studies investigating the role of Bregs with induced AICDA expression during chronic HIV infection and in the pre-tumor microenvironment of AIDS-NHL are warranted. Understanding how these pre-NHL Bregs compare in their regulatory or suppressive functions to Bregs from treatment-naïve or cART-treated PLWH will be important. Defining favorable or unfavorable effects of AICDA^+^ Bregs will provide insight into preventative or therapeutic strategies for precision medicine and/or treatment of pre-AIDS-NHL patients.

### 4.1 Limitations of the study

In this study, we used visit study samples from HIV+ cART-naïve individuals who went on to develop NHL and before their diagnosis. The PBMC samples were specifically from visits conducted by MACS study participants between 1985 and 2002, which only had participants that were homosexual and bisexual men. Future research should include samples from each sex (including gender identity and expression), and from other racial and ethnic groups. A second limitation to this study was the insufficient access to samples by tumor subtype to characterize B-cell populations in the circulation of individuals that went on to develop BL or DLBCL and/or central nervous system (CNS) or non-CNS NHL. A third limitation is the inability to include other antibodies/markers of mature plasma cells [i.e., CD138 or the expression of transcription factors XBP1, IRF4, or BLIMP-1 (regulator of long-lived plasma cells)] and/or other markers that may identify genetic abnormalities in DLBCL (i.e., BCL-2, TP53, and TNFAIP3). It would also be important to define translocation and mutational events in circulating B cells of HIV+ pre-NHL (cART-naïve) samples and identify genetic biomarkers that could predict treatment response in AIDS-NHL patients. The inability to have matched PBMC and serum samples for this specific cohort of pre-lymphoma and HIV+ treatment-naïve samples did not allow the opportunity to examine associations with molecules contributing to chronic immune activation and/or proinflammatory cytokines previously shown to precede the development of AIDS-NHL (14, 36, 38, 57, 70) or serum-derived extracellular vesicles significantly associated with AIDS-NHL risk (71). Future studies will examine associations between B cells and T cells and B cells with M1-like and M2-like monocytes in the circulation of PLWH and in the pre-lymphoma stage.

### 4.2 Conclusions

The unsupervised clustering and immunophenotyping of circulating B cells in HIV+ cART-naïve samples identified pre-lymphoma B-cell phenotypes in this cohort study. We found elevated levels of CD27^+^IgM^+^ B cells, activated memory B-cell populations (CD20^+^CD27^+^CD24^+^CXCR4^+^cMYC^+^ B cells and CD20^+^CD24^+^CXCR4^+^CXCR5^+^ B cells), and CD20^+^CXCR4^hi^AICDA^+^ B cells with malignant and pre-lymphoma phenotypes in the circulation of HIV+ cART-naïve samples at 6 to 36 months before their NHL diagnosis. We also identified a subset of germinal center B-cell-like cells (CD19^-^CD20^+^CXCR4^+^Bcl-6^+^PD-L1^+^cMYC^+^) in the circulation of HIV+ pre-NHL (cART-naïve) samples. Lastly, elevated levels of Bregs expressing aberrant markers and oncogenic features of AICDA and cMYC expression were observed in the circulation of HIV+ pre-NHL (cART-naïve) compared to HIV+ cART-naïve samples. This study provides the notable identification of unique B-cell subsets in the circulation of PLWH that contain pre-malignant and oncogenic characteristics that may drive or contribute to the initiation of pre-tumor B cells in NHL and/or promote lymphomagenesis. Moreover, we identify B-cell populations that are elevated in HIV+ pre-NHL (cART-naïve) samples that may contribute to and promote the development of malignant clones.

## Data Availability

The original contributions presented in the study are included in the article/**Supplementary Material**. Further inquiries can be directed to the corresponding author.
