# Supplementary material for: Characterization of unique B-cell populations in the circulation of people living with HIV prior to non-Hodgkin lymphoma diagnosis
Source: Front Immunol. 2024 Sep 11;15:1441994. doi: 10.3389/fimmu.2024.1441994 (PMC11422120; doi:10.3389/fimmu.2024.1441994)
Supplement: Supplementary file 1 [file DataSheet1.docx]

***Supplementary Material***

**Supplementary Table 1.** Characteristics of HIV+ pre-NHL (cART-naïve) cohort samples.

| **HIV+ pre-NHL**  **(cART-naïve )** | **Interval between PBMC sample collection and lymphoma diagnosis**  **(months)** | **Cancer recorded in the MACS^a^ cancer database, based on SEER^b^ codes** | **Tumor subtype**  **(as defined in**  **biopsy report)** | **Epstein Barr Virus (EBV) status (provided in biopsy report)** |
| --- | --- | --- | --- | --- |
| Case 1 | 12 | NHL, nodal^d^ | N/A^c^ | N/A |
| Case 2 | 28 | NHL, nodal | Malignant, high-grade lymphoma. Small, non-cleaved-cell, non-Burkitt’s type (possibly DLBCL) | N/A |
| Case 3 | 6 | NHL | Malignant, high-grade lymphoma, B-cell immunoblastic type | Positive for EBV latent membrane protein and positive for EBERs by  *in situ* hybridization |
| Case 4 | 12 | NHL | Diagnosed with AIDS dementia -  Extensive CMV encephalopathy /midbrain perivascular lymphoma | N/A |
| Case 5 | 12 | NHL, nodal | High-grade B-cell lymphoma. Small, non-cleaved-cell, non-Burkitt’s type (Stage I)  (possibly DLBCL) | N/A |
| Case 6 | 15 | NHL, nodal | Malignant, large non-cleaved B-cell lymphoma  (possibly DLBCL) | N/A |
| Case 7 | 36 | NHL, extranodal^e^ | N/A | N/A |
| Case 8 | 9 | NHL | Diffuse large B-cell immunoblastic lymphoma  (DLBCL) | EBV positive by detection of early antigen (EA) |
| Case 9 | 12 | NHL | N/A | N/A |
| Case 10 | 27 | NHL, nodal | Diffuse Large B-cell lymphoma (DLBCL) | Positive for EBV latent membrane protein |
| **Median (months)**  **Min (months)**  **Max (months)** | 6 |  | | |
|  | 12 |  |  |  |
|  | 36 |  |  |  |

**^a^MACS:** Multicenter AIDS Cohort Study.

**^b^SEER:** Surveillance, Epidemiology, and End Results (SEER) Program.

**^c^N/A:** Not Available.

**^d^Nodal:** Involving only lymph nodes.

^e^**Extranodal:** Tumor sites other than lymph nodes, spleen, thymus, and the pharyngeal lymphatic ring.

**Supplementary Table 2.** Mass cytometry panel and source of metal-conjugated antibodies.

| **Metal Label** | **Marker** | **Clone** | **Company Supplier** | **Catalog Number** |
| --- | --- | --- | --- | --- |
| **Surface panel** | | | | |
| ^89^Y | CD3 | UCHT1 | BioLegend^a^ | 317302 |
| ^106^Cd | CD14 | M5E2 | BioLegend | 301810 |
| ^110^Cd | HLA-DR | L243 | BioLegend | 307648 |
| ^111^Cd | CD4 | RPA-T4 | BioLegend | 300502 |
| ^113^Cd | CD8a | RPA-T8 | BioLegend | 301002 |
| ^114^Cd | IgG Fc | N/A | Jackson ImmunoResearch Labs^b^ | AB_2337530 |
| ^116^Cd | CD20 | 2H7 | BioLegend | 302302 |
| ^141^Pr | IgM | MHM-88 | BioLegend | 314502 |
| ^142^Nd | CD278 (ICOS) | C398.4A | BioLegend | 313512 |
| ^143^Nd | CD183 (CXCR3) | G025H7 | BioLegend | 353750 |
| ^144^Nd | CD195 (CCR5) | NP-6G4 | Fluidigm/Standard BioTools^c^ | 3144007A |
| ^145^Nd | CD163 | GHI/61 | BioLegend | 333602 |
| ^146^Nd | CD10 | HI10a | BioLegend | 312202 |
| ^147^Sm | CD24 | ML5 | BioLegend | 311127 |
| ^149^Sm | CD19 | HIB19 | BioLegend | 302214 |
| ^150^Nd | CD86 | IT2.2 | Fluidigm/Standard BioTools | 3150020B |
| ^151^Eu | Ig lambda (light chain) | MHL-38 | Fluidigm/Standard BioTools | 3151004B |
| ^153^Eu | CD185 (CXCR5) | RF8B2 | Fluidigm/Standard BioTools | 3142015B |
| ^155^Gd | CD273 (PD-L2) | 24F.10C12 | BioLegend | 329610 |
| ^156^Gd | CD184 (CXCR4) | 12G5 | BioLegend | 306512 |
| ^158^Gd | CD27 | O323 | Fluidigm/Standard BioTools | 302802 |
| ^159^Tb | CD284 (TLR4) | HTA125 | BioLegend | 312808 |
| ^160^Gd | Ig kappa (light chain) | MHK-49 | Fluidigm/Standard BioTools | 3160005B |
| ^164^Dy | CD38 | HIT2 | BioLegend | 303502 |
| ^165^Ho | CD40 | 5C3 | Fluidigm/Standard BioTools | 3165005B |
| ^168^Er | CD154 (CD40L) | 24-31 | Fluidigm/Standard BioTools | 3168006B |
| ^170^Er | CD152 (CTLA-4) | 14D3 | Invitrogen^d^ | 3170005B |
| ^171^Yb | CD28 | CD28.2 | BioLegend | 359602 |
| ^172^Yb | CD279 (PD-1) | EH12.2H7 | BioLegend | 329912 |
| ^173^Yb | CD80 | 2D10.4 | BioLegend | 305246 |
| ^174^Yb | CD71 | OKT | Invitrogen | 14-0719-82 |
| ^175^Lu | CD274 (PD-L1) | 29E.2A3 | BioLegend | 329716 |
| ^209^Bi | CD11b | ICRF44 | Fluidigm/Standard BioTools | 3209003B |
| **Intracellular panel** | | | | |
| ^162^Dy | FoxP3 | PCH101 | Fluidigm/Standard BioTools | 3162011A |
| ^163^Dy | Bcl-6 | K112-91 | Fluidigm/Standard BioTools | 3163012B |
| ^166^Er | IL-10 | JES3-9D7 | Fluidigm/Standard BioTools | 3166008B |
| ^169^Tm | AICDA | 359218 | R&D Systems^e^ | MAB39102 |
| ^176^Yb | c-MYC | 9.00E+10 | Fluidigm/Standard BioTools | 3176012B |
| ^152^Sm | EBV LMP1 | LMPO24 | Novus Biologicals^f^ | NBP2-50383 |
| ^154^Sm | HIV-1 core antigen | KC57 | Beckman Coulter^g^ | IMBULK1B |

^a^BioLegend, San Diego, CA, USA; ^b^Jackson ImmunoResearch Labs, West Grove, PA, USA; ^c^Fluidigm/Standard BioTools, South San Francisco, CA, USA; ^d^Invitrogen, Waltham, MA, USA; ^e^R&D Systems, Minneapolis, MN, USA; ^f^Novus Biologicals, Centennial, CO, USA; ^g^Beckman Coulter, Brea, CA.

CD, cluster of differentiation. HLA-DR, human leukocyte antigen-D-related. IgG Fc, Low affinity immunoglobulin gamma Fc region receptor III-B. IgM, immunoglobulin mu heavy chain. ICOS, inducible T-cell costimulator. CXCR, CXC chemokine receptor. CCR, C-C chemokine receptor. Ig lambda light chain, anti-human immunoglobulin lambda light chain. PD-L2, programmed cell death-ligand 2. TLR4, Toll-like receptor 4. Ig kappa light chain, anti-human immunoglobulin kappa light chain. CTLA-4, Cytotoxic T-lymphocyte protein 4. PD-1, programmed cell death protein 1. PD-L1, programmed death-ligand 1. FoxP3, Forkhead box protein P3. Bcl-6, B-cell lymphoma 6 protein. IL-10, interleukin-10. AICDA, activation-induced cytidine deaminase. cMYC, c-Myc-binding protein. EBV LMP1, Epstein-Barr Virus latent membrane protein 1. HIV-1, human immunodeficiency virus type 1.

**Supplementary Table 3.** Related to **Figure 2**. Phenotypes of CD19^+^ B-cell metaclusters significantly elevated in HIV+ cART-naïve compared to HIV-negative and significant differences in marker expression.

| **Metacluster^a^** | **Group** | **Phenotype** | **Significant marker expression** | ***p*-value^b^** |
| --- | --- | --- | --- | --- |
| **MC42** | HIV-negative | CD20^+^ **CXCR5^+^** IgM^+^ HLA-DR^+^  EBV LMP1+ | CXCR5 elevated in HIV-negative | *p* = 0.001 |
|  | HIV+  cART-naïve | CD20^+^CXCR5^-^ IgM^+^ HLA-DR^+^ |  |  |
| **MC01** | HIV-negative | **CD27^hi^** **CXCR4^+^** CD8^+^ IgM^+^ | CD27 elevated in HIV-negative  CXCR4 elevated in HIV-negative | *p* = 0.040  *p* = 0.009 |
|  | HIV+  cART-naïve | CD27^+^ CXCR4^-^ CD8^+^ IgM^+^ **HLA-DR^+^**  CD8 expression in HIV-negative and HIV+ cART-naïve could represent immune cell complexes of B-cells and non-T-cells (i.e. potentially dendritic cells). | HLA-DR elevated in HIV+ cART-naïve | *p* = 0.030 |
| **MC35** | HIV-negative | CD20^+^ **CD27^+^** CD4^+^ IgM^+^ HLA-DR^+^ | CD27 elevated in HIV-negative | *p* = 0.001 |
|  | HIV+  cART-naïve | CD20^+^ CD27^-^ CD4^+^ IgM^+^ HLA-DR^+^ |  |  |
| **MC19** | HIV-negative | **CD20^hi^** IgM^+^ HLA-DR^+^ | CD20 elevated in HIV-negative | *p* = 0.013 |
|  | HIV+  cART-naïve | CD20^+^ **IgM^hi^** HLA-DR^+^ | IgM elevated in HIV+ cART-naïve | *p* = 0.0002 |
| **MC04** | HIV-negative | CD19^+^ IgM^+^ |  |  |
|  | HIV+  cART-naïve | CD19^+^ IgM^+^ **HLA-DR^+^** | HLA-DR elevated in HIV+ cART-naïve | *p* = 0.014 |
| **MC17** | HIV-negative | CD20^+^ CD27^+^ IgM^+^ HLA-DR^+^ |  |  |
|  | HIV+  cART-naïve | CD20^+^ CD27^-^ **IgM^hi^** HLA-DR^+^ | IgM elevated in HIV+ cART-naïve | *p* =0.0004 |
| **MC06** | HIV-negative | CD20^+^ **CD24^+^** CXCR4^+^ CXCR5^+^  IgM^+^ HLA-DR^+^ | CD24 elevate in HIV-negative | *p* = 0.006 |
|  | HIV+  cART-naïve | CD20^+^ CXCR4^+^ IgM^+^ HLA-DR^+^ |  |  |
| **MC45** | HIV-negative | CD20^+^ CD27^+^ CXCR4^+^ **CXCR5^+^** CD4^+^ **cMYC^+^** IgM^+^ HLA-DR^+^ | CXCR5 elevated in HIV-negative  cMYC elevated in HIV-negative | *p* = 0.001  *p* = 0.044 |
|  | HIV+  cART-naïve | CD20^+^ CD27^-^ CXCR4^+^ CD4^+^  IgM^+^ HLA-DR^+^ |  |  |
| **MC48** | HIV-negative | CD20^+^ **CD27^+^** **CD24^hi^** CXCR4^+^ CXCR5^+^ IgM^+^ HLA-DR^+^ | CD27 elevated in HIV-negative  CD24 elevated in HIV-negative | *p* = 0.002  *p* = 0.021 |
|  | HIV+  cART-naïve | CD20^+^ CD27^-^ CD24^+^ CXCR4^+^ CXCR5^+^ IgM^+^ **HLA-DR^hi^** | HLA-DR elevated in HIV+ cART-naïve | *p* = 0.002 |
| **MC36** | HIV-negative | CD20^+^ CD27^+^ CXCR4^+^ **CXCR5^+^** CD4^+^ CD71^+^ IgM^+^ HLA-DR^+^ | CXCR5 elevated in HIV-negative | *p* = 0.0002 |
|  | HIV+  cART-naïve | CD20^+^ CD27^+^ CXCR4^+^ CXCR5^-^ CD4^+^ CD71^+^ **IgM^hi^** HLA-DR^+^ | IgM elevated in HIV+ cART-naïve | *p* = 0.026 |
| **MC49** | HIV-negative | CD20^+^ CD27^+^ CXCR4^+^ CXCR5^+^ **IgM^hi^** HLA-DR^+^  EBV LMP1+ | IgM elevated in HIV-negative | *p* = 0.009 |
|  | HIV+  cART-naïve | CD20^+^ CD27^+^ CD24^+^ CXCR4^+^ CXCR5^+^ **CD4^+^** **CD71^+^** IgM^+^ HLA-DR^+^ | CD4 elevated in HIV+ cART-naïve  CD71 elevated in HIV+ cART-naïve | *p* = 0.038  *p* = 0.022 |
| **MC15** | HIV-negative | CD20^+^ CD27^+^ CXCR4^+^ **CXCR5^hi^**  IgM^+^ HLA-DR^+^ | CXCR5 elevated in HIV-negative | *p* = 0.022 |
|  | HIV+  cART-naïve | CD20^+^ CD27^+^ CXCR4^+^ CXCR5^+^  **IgM^hi^** HLA-DR^+^ | IgM elevated in HIV+ cART-naïve | *p* = 0.010 |
| **MC50** | HIV-negative | N/A |  |  |
|  | HIV+  cART-naive | CD20^+^ CXCR4^+^ cMYC^+^ IgM^+^  HLA-DR^+^ |  |  |
| **MC18** | HIV-negative | CXCR4^+^ CD4^+^ **FoxP3^+^** IgM^+^ HLA-DR^+^ | FoxP3 elevated in HIV-negative | *p* = 0.021 |
|  | HIV+  cART-naïve | CXCR4^+^ CD4^+^ IgM^+^ HLA-DR^+^  EBV LMP1+ |  |  |
| **MC02** | HIV-negative | CD10^+^ CD27^+^ CD24^+^ CD40^+^ CD86^+^ CXCR4^+^ CCR5^+^ ICOS^+^ **CD4^+^**  IgM^+^ HLA-DR^+^ | CD4 elevated in HIV-negative | *p* = 0.018 |
|  | HIV+  cART-naïve | CD10^+^ CD27^-^ CD24^+^ CD40^+^ CD86^+^ CXCR4^+^ CCR5^+^ **CD8^+^** IgM^+^ HLA-DR^+^  EBV LMP1+ | CD8 elevated in HIV+ cART-naïve | *p* = 0.007 |
| **MC03** | HIV-negative | CD27^+^ CD28^+^ CXCR4^+^ CD4^+^  IgM^+^ HLA-DR^+^ |  |  |
|  | HIV+  cART-naïve | CD27^+^ CD28^+^ CXCR4^+^ CD4^+^  IgM^+^ HLA-DR^+^ |  |  |
| **MC11** | HIV-negative | CD10^+^ CD27^+^ CXCR4^+^ CXCR3^+^ CCR5^+^ ICOS^+^ CD4^+^ IgM^+^ HLADR^+^ |  |  |
|  | HIV+  cART-naïve | CD10^+^ CD27^-^ CXCR4^+^ CXCR3^-^  CCR5^+^ ICOS^-^ CD4^+^ IgM^+^ HLA-DR^+^ |  |  |
| **MC25** | HIV-negative | CD20^+^ CXCR4^+^ IgM^+^ HLA-DR^+^ |  |  |
|  | HIV+  cART-naïve | CD20^+^ CXCR4^+^ IgM^+^ HLA-DR^+^ |  |  |

**^a^Metacluster:** Provided are the 18 CD19^+^ B-cell metaclusters significantly elevated in HIV+ cART-naïve compared to HIV-negative. Metaclusters are organized by highest to lowest order of significant *p*-values (equal to or greater than -log10 (*p*-value of 0.05) or 1.30) as provided in **Figure 2A**.

**^b^*p*-value:** Significant *p*-value determined when comparing marker expression. Unpaired, non-parametric Mann-Whitney test.

**Supplementary Table 4.** Related to **Figure 3 and Figure 3C**. Phenotypes of CD19^+^ B-cell metaclusters significantly elevated in HIV+ pre-NHL (cART-naïve) compared to HIV+ cART-naïve and significant differences in marker expression.

| **Metacluster^a^** | **Group** | **Phenotype** | **Significant marker expression** | ***p*-value^b^** |
| --- | --- | --- | --- | --- |
| **MC44** | HIV-negative^c^ | **CD20^hi^** **CD27^hi^** CXCR4^+^ CXCR5^+^ CD4^+^  IgM^+^ HLA-DR^+^  EBV LMP1^+^ | CD20 elevated in HIV-negative vs. HIV+ pre-NHL (cART-naïve)  CD27 elevated in HIV-negative vs. HIV+ cART-naïve  CD27 elevated in HIV-negative vs. HIV+ pre-NHL  (cART-naïve) | *p* = 0.017  *p =* 0.002  *p* = 0.004 |
|  | HIV+  cART-naïve | **CD20^hi^** CD27^+^ CXCR4^+^ CXCR5^+^  CD24^+^ IgM^+^ HLA-DR^+^ | CD20 elevated in HIV+ cART-naïve vs. HIV+ pre-NHL (cART-naïve) | *p* = 0.036 |
|  | HIV+ pre-NHL (cART-naïve) | CD20^+^ CD27^+^ CD24^+^ CXCR4^+^ CXCR5^+^  IgM^+^ HLA-DR^+^ |  |  |
| **MC48** | HIV-negative | CD20^+^ **CD27^+^** **CD24^hi^** CXCR4^+^ CXCR5^+^ IgM^+^ HLA-DR^+^ | CD27 elevated in HIV-negative vs. HIV+ cART-naïve  CD27 elevated in HIV-negative vs. HIV+ pre-NHL (cART-naïve)  CD24 elevated in HIV-negative vs. HIV+ cART-naïve | *p* = 0.002  *p* = 0.002  *p* = 0.021 |
|  | HIV+  cART-naïve | CD20^+^ CD27^-^ CD24^+^ CXCR4^+^ CXCR5^+^  IgM^+^ **HLA-DR^+^** | HLA-DR elevated in HIV+ cART-naïve vs. HIV-negative | *p* = 0.002 |
|  | HIV+ pre-NHL (cART-naïve) | CD20^+^ CD27^-^ CD24^+^ CXCR4^+^ CXCR5^+^  IgM^+^ HLA-DR^+^ |  |  |
| **MC39** | HIV-negative | CD20^+^ **CD27^+^** CXCR4^+^ CXCR5^+^  IgM^+^ HLA-DR^+^ | CD27 elevated in HIV-negative vs. HIV+ cART-naïve  CD27 elevated in HIV-negative vs. HIV+ pre-NHL (cART-naïve) | *p* = 0.014  *p* = 0.003 |
|  | HIV+  cART-naïve | CD20^+^ CD27^-^ CXCR4^+^ CXCR5^-^  IgM^+^ HLA-DR^+^ |  |  |
|  | HIV+ pre-NHL (cART-naïve) | CD20^+^ CD27^-^ CXCR4^+^ CXCR5^-^  IgM^+^ HLA-DR^+^ |  |  |
| **MC37** | HIV-negative | CD20^+^ CXCR4^+^ **CXCR5^+^** IgM^+^  HLA-DR^+^ | CXCR5 elevated in HIV-negative vs. HIV+ pre-NHL (cART-naïve) | *p* = 0.003 |
|  | HIV+  cART-naïve | **CD20^+^** CXCR4^+^ **CXCR5^+^** IgM^+^  HLA-DR^+^ | CD20 elevated in HIV+ cART-naïve vs. HIV-negative  CXCR5 elevated in HIV+ cART-naïve vs. HIV+ pre-NHL (cART-naïve) | *p* = 0.019  *p* = 0.019 |
|  | HIV+ pre-NHL (cART-naïve) | CD20^+^ CXCR4^+^ CXCR5^-^ IgM^+^  HLA-DR^+^ |  |  |
| **MC47** | HIV-negative | CD20^+^ **CD27^hi^** CD10^+^ CD24^+^ CXCR4^+^ cMYC^+^ IgM^+^ HLA-DR^+^ | CD27 elevated in HIV-negative vs. HIV+ cART-naïve  CD27 elevated in HIV-negative vs. HIV+ pre-NHL (cART-naïve) | *p* = 0.008  *p* = 0.0003 |
|  | HIV+  cART-naïve | CD20^+^ CD27^+^ CD10^+^ CD24^+^ CXCR4^+^ cMYC^+^ IgM^+^ **HLA-DR^hi^** | HLA-DR elevated in HIV+ cART-naïve vs. HIV+ pre-NHL (cART-naïve) | *p* = 0.048 |
|  | HIV+ pre-NHL (cART-naïve) | CD20^+^ CD27^+^ CD10^+^ CD24^+^ CXCR4^+^ cMYC^+^ IgM^+^ HLA-DR^+^ |  |  |
| **MC10** | HIV-negative | CD27^+^ CXCR4^+^ IgM^+^ HLA-DR^+^ |  |  |
|  | HIV+  cART-naïve | CD27^+^ CXCR4^-^ IgM^+^ HLA-DR^+^ |  |  |
|  | HIV+ pre-NHL (cART-naïve) | CD27^+^ CXCR4^-^ IgM^+^ HLA-DR^-^ |  |  |

**^a^Metacluster:** Provided are the 6 CD19^+^ B-cell metaclusters significantly elevated in HIV+ pre-NHL (cART-naïve) compared to HIV+ cART-naïve. Metaclusters are organized by highest to lowest order of significant *p*-values (equal to or greater than -log10 (*p*-value of 0.05) or 1.30) as provided in **Figure 3A**.

**^b^*p*-value:** Significant *p*-value determined when comparing marker expression. Unpaired, non-parametric Mann-Whitney test.

**^c^HIV-negative:** The CD19^+^ B-cell phenotypes of each metacluster for HIV-negative are provided in order to compare with the phenotypes observed in HIV+ cART-naïve and HIV+ pre-NHL (cART-naïve) samples.

**Supplementary Table 5.** Related to **Figure 3 and Figure 3D**. Phenotypes of CD19^+^ B-cell metaclusters significantly elevated in HIV+ cART-naïve compared to HIV+ pre-NHL (cART-naïve) and significant differences in marker expression.

| **Metacluster^a^** | **Group** | **Phenotype** | **Significant marker expression** | ***p*-value^b^** |
| --- | --- | --- | --- | --- |
| **MC31** | HIV-negative^c^ | CD20^+^ CXCR4^+^ CXCR5^+^ IgM^+^  HLA-DR^+^ |  |  |
|  | HIV+  cART-naïve | CD20^+^ CXCR4^+^ CXCR5^+^ IgM^+^  HLA-DR^+^ |  |  |
|  | HIV+ pre-NHL (cART-naïve) | CD20^+^ CXCR4^+^ CXCR5^+^ IgM^+^  HLA-DR^+^ |  |  |
| **MC08** | HIV-negative | CD20^+^ **CD27^hi^** CD24^+^ CXCR4^+^ **CXCR5^hi^** IgM^+^ HLA-DR^+^ | CD27 elevated in HIV-negative vs. HIV+ cART-naïve  CXCR5 elevated in HIV-negative vs. HIV+ cART-naïve  CXCR5 elevated in HIV-negative vs. HIV+ pre-NHL (cART-naïve) | *p* = 0.013  *p* = 0.0006  *p* = 0.0002 |
|  | HIV+  cART-naïve | CD20^+^ CD27^+^ CD24^+^ CXCR4^+^ **CXCR5^+^** IgM^+^ HLA-DR^+^ | CXCR5 elevated in HIV+ cART-naïve vs. HIV+ pre-NHL (cART-naïve) | *p* = 0.0240 |
|  | HIV+ pre-NHL (cART-naïve) | CD20^+^ CD27^+^ CD24^+^ CXCR4^+^ CXCR5^+^ IgM^+^ HLA-DR^+^ |  |  |
| **MC07** | HIV-negative | CD20^+^ CD24^-^ CXCR4^+^ **CXCR5^hi^**  IgM^+^ HLA-DR^+^ | CXCR5 elevated in HIV-negative vs. HIV+ cART-naïve  CXCR5 elevated in HIV-negative vs. HIV+ pre-NHL (cART-naïve) | *p* < 0.0001  *p* = 0.0002 |
|  | HIV+  cART-naïve | CD20^+^ CD24^-^ CXCR4^+^ CXCR5^+^  IgM^+^ HLA-DR^+^ |  |  |
|  | HIV+ pre-NHL (cART-naïve) | CD20^+^ CD24^+^ CXCR4^+^ CXCR5^+^  **IgM^hi^** HLA-DR^+^ | IgM elevated in HIV+ pre-NHL (cART-naïve) vs. HIV-negative  IgM elevated in HIV+ pre-NHL (cART-naïve) vs. HIV+ cART-naïve | *p* = 0.002  *p* = 0.023 |
| **MC20** | HIV-negative | CD20^+^ CD24^-^ CXCR4^+^ **CXCR5^hi^**  IgM^+^ HLA-DR^+^ | CXCR5 elevated in HIV-negative vs. HIV+ cART-naïve  CXCR5 elevated in HIV-negative vs. HIV+ pre-NHL (cART-naïve) | *p* = 0.0001  *p* < 0.0001 |
|  | HIV+  cART-naïve | CD20^+^ CD24^-^ CXCR4^+^ CXCR5^+^  IgM^+^ HLA-DR^+^ |  |  |
|  | HIV+ pre-NHL (cART-naïve) | CD20^+^ CD24^+^ CXCR4^+^ **CXCR5^hi^**  IgM^+^ HLA-DR^+^ | CXCR5 elevated in HIV+ pre-NHL (cART-naïve) vs. HIV+ cART-naïve | *p* = 0.013 |
| **MC28** | HIV-negative | CD20^+^ **CD27^hi^** CD24^+^ CXCR4^+^ **CXCR5^hi^** IgM^+^ HLA-DR^+^ | CD27 elevated in HIV-negative vs. HIV+ cART-naïve  CXCR5 elevated in HIV-negative vs. HIV+ cART-naïve  CXCR5 elevated in HIV-negative vs. HIV+ pre-NHL (cART-naïve) | *p* = 0.031  *p* = 0.0001  *p* = 0.001 |
|  | HIV+  cART-naïve | CD20^+^ CD27^+^ CD24^+^ CXCR4^+^ CXCR5^+^ IgM^+^ HLA-DR^+^ |  |  |
|  | HIV+ pre-NHL (cART-naïve) | CD20^+^ CD27^+^ **CD24^hi^** CXCR4^+^ CXCR5^+^ IgM^+^ HLA-DR^+^ | CD24 elevated in HIV+ pre-NHL (cART-naïve) vs. HIV+ cART-naïve | *p* = 0.009 |
| **MC49** | HIV-negative | CD20^+^ CD27^+^ CXCR4^+^ **CXCR5^+^** CD4^-^ CD71^-^ **IgM^hi^** HLA-DR^+^  EBV LMP1+ | IgM elevated in HIV-negative vs. HIV+ cART-naïve  IgM elevated in HIV-negative vs. HIV+ pre-NHL (cART-naïve)  CXCR5 elevated in HIV-negative vs. HIV+ pre-NHL (cART-naïve) | *p* = 0.009  *p* = 0.002  *p* = 0.009 |
|  | HIV+  cART-naïve | CD20^+^ CD27^+^ CXCR4^+^ CXCR5^+^ **CD4^+^ CD71^+^** IgM^+^ HLA-DR^+^ | CD4 elevated in HIV+ cART-naïve vs. HIV-negative  CD4 elevated in HIV+ cART-naïve vs. HIV+ pre-NHL (cART-naïve)  CD71 elevated in HIV+ cART-naïve vs. HIV-negative | *p* = 0.038  *p* = 0.039  *p* = 0.022 |
|  | HIV+ pre-NHL (cART-naïve) | CD20^+^ CD27^+^ CXCR4^+^ CXCR5^-^ CD4^-^ CD71^-^ IgM^+^ HLA-DR^+^  EBV LMP1+ |  |  |
| **MC21** | HIV-negative | CD20^+^ CD27^+^ CD24^+^ CD40^+^ CXCR4^+^ **CXCR5^hi^** IgM^+^  HLA-DR^+^ | CXCR5 elevated in HIV-negative vs. HIV+ cART-naïve | *p* = 0.010 |
|  | HIV+  cART-naïve | CD20^+^ CD27^+^ CD24^+^ CD40^+^ CXCR4^+^ CXCR5^+^ IgM^+^ HLA-DR^+^ |  |  |
|  | HIV+ pre-NHL (cART-naïve) | CD20^+^ **CD27^hi^** CD24^+^ CD40^+^ CXCR4^+^ CXCR5^+^ **FoxP3^+^** **cMYC^+^ AICDA^+^** IgM^+^ HLA-DR^+^ | CD27 elevated in HIV+ pre-NHL (cART-naïve) vs. HIV+ cART-naive | *p* = 0.020 |
| **MC14** | HIV-negative | CD20^+^ CD27^+^ CD24^+^ CD40^+^ **CXCR4^+^** **CXCR5^hi^** IgM^+^  HLA-DR^+^ | CXCR4 elevated in HIV-negative vs. HIV+ pre-NHL (cART-naïve)  CXCR5 elevated in HIV-negative vs. HIV+ cART-naïve  CXCR5 elevated in HIV-negative vs. HIV+ pre-NHL (cART-naïve) | *p* = 0.043  *p* = 0.003  *p* = 0.013 |
|  | HIV+  cART-naïve | CD20^+^ CD27^+^ CD24^+^ CD40^+^ CXCR4^+^ CXCR5^+^ IgM^+^ HLA-DR^+^ |  |  |
|  | HIV+ pre-NHL (cART-naïve) | CD20^+^ CD27^+^ CD24^+^ CD40^+^ CXCR4^+^ CXCR5^+^ **cMYC^+^ AICDA^+^** IgM^+^ HLA-DR^+^ |  |  |
| **MC29** | HIV-negative | CD20^+^ CD27^+^ **CXCR5^hi^** CD4^+^ CD71^+^ IgM^+^ HLA-DR^+^  EBV LMP1+ | CXCR5 elevated in HIV-negative vs. HIV+ cART-naïve  CXCR5 elevated in HIV-negative vs. HIV+ pre-NHL (cART-naïve) | *p* = 0.025  *p* = 0.025 |
|  | HIV+  cART-naïve | CD20^+^ CD27^+^ CXCR5^+^ CD4^+^ CD71^+^ IgM^+^ HLA-DR^+^  EBV LMP1+ |  |  |
|  | HIV+ pre-NHL (cART-naïve) | CD20^+^ CD27^+^ CXCR5^-^ CD4^+^ CD71^+^ **IgM^+^** HLA-DR^+^  EBV LMP1+ | IgM elevated in HIV+ pre-NHL (cART-naïve) vs. HIV-negative | *p* = 0.001 |
| **MC23** | HIV-negative | CD20^+^ CD27^+^ CXCR4^+^ **CXCR5^hi^** CD4^+^ CD8^-^ CD71^-^ IgM^+^  **HLA-DR^hi^** | HLA-DR elevated in HIV-negative vs. HIV+ cART-naïve  CXCR5 elevated in HIV-negative vs. HIV+ cART-naïve | *p* = 0.027  *p* = 0.023 |
|  | HIV+  cART-naïve | CD20^+^ CD27^+^ CXCR4^+^ CXCR5^+^ CD4^+^ **CD8^+^** CD71^-^ IgM^+^  HLA-DR^+^  EBV LMP1^+^ | CD8 elevated in HIV+ cART-naïve vs. HIV-negative | *p* = 0.0003 |
|  | HIV+ pre-NHL (cART-naïve) | CD20^+^ CD27^+^ CXCR4^+^ CXCR5^+^ CD4^+^ **CD8^+^** **CD71^+^** IgM^+^  HLA-DR^+^ | CD8 elevated in HIV+ pre-NHL (cART-naïve) vs. HIV-negative  CD71 elevated in HIV+ pre-NHL (cART-naïve) vs. HIV-negative  CD71 elevated in HIV+ pre-NHL (cART-naïve) vs. HIV+ cART-naïve | *p* = 0.024  *p* = 0.007  *p* = 0.041 |

**^a^Metacluster:** Provided are the 10 CD19^+^ B-cell metaclusters significantly elevated in HIV+ cART-naïve compared to HIV+ pre-NHL (cART-naïve). Metaclusters are organized by highest to lowest order of significant *p*-values (equal to or greater than -log10 (*p*-value of 0.05) or 1.30) as provided in **Figure 3A**.

**^b^*p*-value:** Significant *p*-value determined when comparing marker expression. Unpaired, non-parametric Mann-Whitney test.

**^c^HIV-negative:** The CD19^+^ B-cell phenotypes of each metacluster for HIV-negative are provided in order to compare with the phenotypes observed in HIV+ cART-naïve and HIV+ pre-NHL (cART-naïve) samples.

**Supplementary Table 6.** Related to **Figure 4**. Phenotypes of CD19^+^ B-cell metaclusters significantly elevated in HIV+ pre-NHL (cART-naïve) compared to HIV-negative and significant differences in marker expression.

| **Metacluster^a^** | **Group** | **Phenotype** | **Significant marker expression** | ***p*-value^b^** |
| --- | --- | --- | --- | --- |
| **MC42** | HIV-negative | CD20^+^ **CXCR5^+^** IgM^+^ HLA-DR^+^  EBV LMP1+ | CXCR5 elevated in HIV-negative | *p* = 0.001 |
|  | HIV+ pre-NHL  (cART-naïve) | CD20^+^ CXCR5^-^ IgM^+^ HLA-DR^+^ |  |  |
| **MC01** | HIV-negative | **CD27^hi^** CXCR4^+^ CD8^+^ IgM^+^  HLA-DR^-^ | CD27 elevated in HIV-negative | *p* = 0.007 |
|  | HIV+ pre-NHL  (cART-naïve) | CD27^+^ CXCR4^-^ CD8^+^ **IgM^hi^**  HLA-DR^+^  CD8 expression in HIV-negative and HIV+ cART-naïve could represent immune cell complexes of B-cells and non-T-cells (i.e. potentially dendritic cells). | IgM elevated in HIV+ pre-NHL (cART-naïve) | *p* = 0.013 |
| **MC48** | HIV-negative | CD20^+^ **CD27^+^** CD24^+^ CXCR4^+^ CXCR5^+^ IgM^+^ HLA-DR^+^ | CD27 elevated in HIV-negative | *p* = 0.002 |
|  | HIV+ pre-NHL  (cART-naïve) | CD20^+^ CD27^-^ CD24^+^ CXCR4^+^ CXCR5^+^ IgM^+^ **HLA-DR^hi^** | HLA-DR elevated in HIV+ pre-NHL (cART-naïve) | *p* = 0.001 |
| **MC35** | HIV-negative | **CD20^hi^ CD27^+^** CD4^+^ IgM^+^  HLA-DR^+^ | CD20 elevated in HIV-negative  CD27 elevated in HIV-negative | *p* = 0.021  *p* = 0.0003 |
|  | HIV+ pre-NHL  (cART-naïve) | CD20^+^ CD27^-^ CD4^-^ IgM^+^ HLA-DR^+^ |  |  |
| **MC04** | HIV-negative | CD19^+^ IgM^+^ HLA-DR^-^ |  |  |
|  | HIV+ pre-NHL  (cART-naïve) | CD19^+^ **IgM^hi^** **HLA-DR^+^** | IgM elevated in HIV+ pre-NHL (cART-naïve)  HLA-DR elevated in HIV+ pre-NHL (cART-naïve) | *p* = 0.010  *p* = 0.050 |
| **MC44** | HIV-negative | **CD20^hi^** **CD27^hi^** CXCR4^+^ CXCR5^+^ CD4^+^ CD24^-^ IgM^+^ HLA-DR^+^  EBV LMP1^+^ | CD20 elevated in HIV-negative vs. HIV+ pre-NHL (cART-naïve)  CD27 elevated in HIV-negative  vs. HIV+ pre-NHL (cART-naïve) | *p* = 0.017  *p* = 0.004 |
|  | HIV+ pre-NHL  (cART-naïve) | CD20^+^ CD27^+^ CXCR4^+^ CXCR5^+^  CD4^-^ CD24^+^ IgM^+^ HLA-DR^+^ |  |  |
| **MC47** | HIV-negative | CD20^+^ **CD27^hi^** CD10^+^ CD24^+^ CXCR4^+^ cMYC^+^ IgM^+^ HLA-DR^+^ | CD27 elevated in HIV-negative | *p* = 0.0003 |
|  | HIV+ pre-NHL  (cART-naïve) | CD20^+^ CD27^+^ CD10^+^ CD24^+^ CXCR4^+^ cMYC^+^ IgM^+^ HLA-DR^+^ |  |  |
| **MC50** | HIV-negative | N/A |  |  |
|  | HIV+ pre-NHL  (cART-naïve) | CD20^+^ CD86^+^ IgM^+^ HLA-DR^+^ |  |  |
| **MC39** | HIV-negative | CD20^+^ **CD27^+^** CXCR4^+^ CXCR5^+^  IgM^+^ HLA-DR^+^ | CD27 elevated in HIV-negative | *p* = 0.003 |
|  | HIV+ pre-NHL  (cART-naïve) | CD20^+^ CD27^-^ CXCR4^+^ CXCR5^-^  IgM^+^ HLA-DR^+^ |  |  |
| **MC45** | HIV-negative | CD20^+^ CD27^+^ CXCR4^+^ **CXCR5^+^** CD4^+^ cMYC^+^ IgM^+^ HLA-DR^+^ | CXCR5 elevated in HIV-negative | *p* = 0.003 |
|  | HIV+ pre-NHL  (cART-naïve) | CD20^+^ CD27^-^ CXCR4^+^ CXCR5^-^ CD4^+^ IgM^+^ HLA-DR^+^  EBV LMP1+ |  |  |
| **MC03** | HIV-negative | CD27^+^ CD28^+^ CXCR4^+^ CD4^+^ IgM^+^ |  |  |
|  | HIV+ pre-NHL  (cART-naïve) | CD27^+^ CD28^+^ CXCR4^+^ CD4^+^ IgM^+^ |  |  |
| **MC11** | HIV-negative | CD10^+^ **CD27^+^** CXCR4^+^ CXCR3^+^ CCR5^+^ ICOS^+^ CD4^+^ IgM^+^ HLADR^+^  EBV LMP1+ | CD27 elevated in HIV-negative | *p* = 0.048 |
|  | HIV+ pre-NHL  (cART-naïve) | CD10^+^ CD27^-^ CXCR4^+^ CXCR3^-^ CCR5^-^ ICOS^-^ CD4^+^ IgM^+^ HLA-DR^+^ |  |  |
| **MC10** | HIV-negative | CD27^+^ CXCR4^+^ IgM^+^ HLA-DR^+^ |  |  |
|  | HIV+ pre-NHL  (cART-naïve) | CD27^+^ CXCR4^-^ IgM^+^ HLA-DR^-^ |  |  |
| **MC25** | HIV-negative | CD20^+^ CXCR4^+^ IgM^+^ HLA-DR^+^ |  |  |
|  | HIV+ pre-NHL  (cART-naïve) | CD20^+^ CXCR4^+^ IgM^+^ HLA-DR^+^ |  |  |
| **MC51** | HIV-negative | CD20^+^ **CXCR5^hi^** IgM^+^ HLA-DR^+^ | CXCR5 elevated in HIV-negative | *p* = 0.002 |
|  | HIV+ pre-NHL  (cART-naïve) | CD20^+^ CXCR5^+^ IgM^+^ HLA-DR^+^ |  |  |
| **MC52** | HIV-negative | CD20^+^ **CD27^hi^** CD24^+^ CXCR4^+^ **CXCR5^hi^** **FoxP3^+^** cMYC^+^  IgM^+^ HLA-DR^+^ | CD27 elevated in HIV-negative  CXCR5 elevated in HIV-negative  FoxP3 elevated in HIV-negative | *p* = 0.003  *p* = 0.007  *p* = 0.021 |
|  | HIV+ pre-NHL  (cART-naïve) | CD20^+^ CD27^+^ CD24^+^ CXCR4^+^ CXCR5^+^ CD4^+^ cMYC^+^  IgM^+^ HLA-DR^+^ |  |  |
| **MC02** | HIV-negative | CD10^+^ CD27^+^ CD24^+^ CD40^+^ CD86^+^ CXCR4^+^ CCR5^+^ **ICOS^+^** CD4^+^ IgM^+^ HLA-DR^+^ | ICOS elevated in HIV-negative | *p* = 0.005 |
|  | HIV+ pre-NHL  (cART-naïve) | CD10^-^ CD27^+^ CD86^+^ CXCR4^+^ ICOS^-^ CD8^+^ IgM^+^  EBV LMP1+ |  |  |
| **MC06** | HIV-negative | CD20^+^ **CD24^+^** CXCR4^+^ CXCR5^+^  IgM^+^ HLA-DR^+^ | CD24 elevated in HIV-negative | *p* = 0.028 |
|  | HIV+ pre-NHL  (cART-naïve) | CD20^+^ CD24^-^ CXCR4^+^ CXCR5^-^  IgM^+^ HLA-DR^+^ |  |  |
| **MC19** | HIV-negative | **CD20^hi^** IgM^+^ HLA-DR^+^ | CD20 elevated in HIV-negative | *p* = 0.009 |
|  | HIV+ pre-NHL  (cART-naïve) | CD20^+^ **IgM^hi^** HLA-DR^+^ | IgM elevated in HIV+ pre-NHL (cART-naïve) | *p* < 0.0001 |
| **MC37** | HIV-negative | CD20^+^ CXCR4^+^ **CXCR5^+^**  IgM^+^ HLA-DR^+^ | CXCR5 elevated in HIV-negative | *p* = 0.003 |
|  | HIV+ pre-NHL  (cART-naïve) | CD20^+^ CXCR4^+^ CXCR5^-^  IgM^+^ HLA-DR^+^ |  |  |
| **MC18** | HIV-negative | CXCR4^+^ CD4^+^ FoxP3^+^ IgM^+^  **HLA-DR^hi^** | HLA-DR elevated in HIV-negative | *p* = 0.032 |
|  | HIV+ pre-NHL  (cART-naïve) | CXCR4^+^ CD4^+^ **IgM^hi^** HLA-DR^+^ | IgM elevated in HIV+ pre-NHL (cART-naïve) | *p* = 0.006 |

**^a^Metacluster:** Provided are the 21 CD19^+^ B-cell metaclusters significantly elevated in HIV+ pre-NHL (cART-naïve) compared to HIV-negative. Metaclusters are organized by highest to lowest order of significant *p*-values (equal to or greater than -log10 (*p*-value of 0.05) or 1.30) as provided in **Figure 4A**.

**^b^*p*-value:** Significant *p*-value determined when comparing marker expression. Unpaired, non-parametric Mann-Whitney test.

**Supplementary Table 7.** Related to **Figure 5**. Phenotypes of CD20^+^CXCR4^hi^ metaclusters significantly elevated in HIV+ pre-NHL (cART-naïve) compared to HIV+ cART-naïve and significant differences in marker expression.

| **CD20^+^CXCR4^hi^ metaclusters elevated in HIV+ pre-NHL (cART-naïve)^a^** | | | | |
| --- | --- | --- | --- | --- |
| **Metacluster** | **Group** | **Phenotype** | **Significant marker expression** | ***p*-value^b^** |
| **MC23** | HIV-negative^c^ | CD20^+^ CXCR4^hi^ **CD27^+^** CD24^+^ **CXCR5^hi^** CD40^-^ CD4^-^ IgM^+^  HLA-DR^+^ | CD27 elevated in HIV-negative  vs. HIV+ cART-naïve  CXCR5 elevated in HIV-negative  vs. HIV+ cART-naïve  CXCR5 elevated in HIV-negative vs. HIV+ pre-NHL (cART-naïve) | *p* = 0.004  *p* < 0.001  *p* < 0.001 |
|  | HIV+  cART-naïve | CD20^+^ CXCR4^hi^ CD27^-^ CD24^+^ CXCR5^+^ **CD40^+^** **CD4^hi^** cMYC^+^  IgM^+^ HLA-DR^+^ | CD40 elevated in HIV+ cART-naïve vs. HIV-negative  CD4 elevated in HIV+ cART-naïve vs. HIV-negative  CD4 elevated in HIV+ cART-naïve vs. HIV+ pre-NHL (cART-naïve) | *p* = 0.047  *p* = 0.016  *p* = 0.017 |
|  | HIV+ pre-NHL (cART-naïve) | CD20^+^ CXCR4^hi^ CD27^-^ CD24^+^ CXCR5^+^ **CD40^+^** **CD4^+^** AICDA^+^  IgM^+^ HLA-DR^+^ | CD40 elevated in HIV pre-NHL (cART-naïve) vs. HIV-negative  CD4 elevated in HIV pre-NHL (cART-naïve) vs. HIV-negative | *p* = 0.049  *p* = 0.017 |
| **MC36** | HIV-negative | CD20^+^ CXCR4^hi^ CD10^+^ **CD27^+^** CD24^+^  IgM^+^ HLA-DR^+^ | CD27 elevated in HIV-negative vs. HIV-cART-naïve  CD27 elevated in HIV-negative vs. HIV+ pre-NHL (cART-naïve) | *p* = 0.004  *p* = 0.006 |
|  | HIV+  cART-naïve | CD20^+^ CXCR4^hi^ CD10^+^ CD27^-^ CD24^+^ CXCR5^+^ IgM^+^ HLA-DR^+^ |  |  |
|  | HIV+ pre-NHL (cART-naïve) | CD20^+^ CXCR4^hi^ CD10^+^ CD27^-^ CD24^+^CXCR5^-^ IgM^+^ **HLA-DR^hi^** | HLA-DR elevated in HIV+ pre-NHL (cART-naïve) vs. HIV+ cART-naïve | *p* = 0.043 |
| **CD20^+^CXCR4^hi^ metaclusters elevated in HIV+ cART-naïve^d^** | | | | |
| **Metacluster** | **Group** | **Phenotype** | **Marker expression** | ***p*-value^b^** |
| **MC28** | HIV-negative | **CD20^hi^** CXCR4^hi^ CD24^+^ CXCR4^+^ **CXCR5^hi^** IgM^+^ **HLA-DR^+^** | CD20 elevated in HIV-negative vs. HIV+ cART naïve  CD20 elevated in HIV-negative vs. HIV+ pre-NHL (cART-naïve)  CXCR5 elevated in HIV-negative  vs. HIV+ cART-naïve  CXCR5 elevated in HIV-negative vs. HIV+ pre-NHL (cART-naïve)  HLA-DR elevated in HIV-negative vs. HIV+ pre-NHL (cART-naïve) | *p* = 0.034  *p* = 0.008  *p* = 0.011  *p* = 0.001  *p* = 0.047 |
|  | HIV+  cART-naïve | CD20^+^ CXCR4^hi^ CD24^+^ CXCR4^+^ **CXCR5^hi^** IgM^+^ HLA-DR^+^ | CXCR5 elevated in HIV+ cART-naïve vs. HIV+ pre-NHL (cART-naïve) | *p* = 0.013 |
|  | HIV+ pre-NHL (cART-naïve) | CD20^+^ CXCR4^hi^ CD24^+^ CXCR4^+^ CXCR5^+^ IgM^+^ HLA-DR^+^ |  |  |
| **MC25** | HIV-negative | CD20^+^ CXCR4^hi^ **CXCR5^hi^ CD40^+^** CD4^-^ IgM^+^ HLA-DR^+^ | CXCR5 elevated in HIV-negative vs. HIV+ cART-naïve  CXCR5 elevated in HIV-negative  vs. HIV+ pre-NHL (cART-naïve)  CD40 elevated in HIV-negative vs. HIV+ pre-NHL (cART-naïve) | *p* = 0.002  *p* = 0.042  *p* = 0.001 |
|  | HIV+  cART-naïve | CD20^+^ CXCR4^hi^ CXCR5^+^ **CD40^+^** **CD4^+^** IgM^+^ HLA-DR^+^ | CD4 elevated in HIV+ cART-naïve vs. HIV-negative  CD40 elevated in HIV+ cART-naïve vs. HIV+ pre-NHL (cART-naïve) | *p* = 0.010  *p* = 0.001 |
|  | HIV+ pre-NHL (cART-naïve) | CD20^+^ CXCR4^hi^ CXCR5^+^ CD40^-^CD4^+^ IgM^+^ HLA-DR^+^ |  |  |
| **MC19** | HIV-negative | CD20^+^ CXCR4^hi^  **CD24^+^** **CXCR5^hi^** CD40^-^ CD4^-^ IgM^+^  HLA-DR^+^ | CD24 elevated in HIV-negative vs. HIV+ pre-NHL (cART-naïve)  CXCR5 elevated in HIV-negative vs. HIV+ cART-naïve  CXCR5 elevated in HIV-negative vs. HIV+ pre-NHL (cART-naïve) | *p* = 0.021  *p* = 0.001  *p* = 0.001 |
|  | HIV+  cART-naïve | CD20^+^ CXCR4^hi^ **CD24^+^** CXCR5^+^ CD40^+^ CD4^+^ IgM^+^ HLA-DR^+^ | CD24 elevated in HIV+ cART-naïve vs. HIV+ pre-NHL (cART-naïve) | *p* = 0.004 |
|  | HIV+ pre-NHL (cART-naïve) | CD20^+^ CXCR4^hi^ CD24^-^ CXCR5^+^  CD40^-^ CD4^-^ IgM^+^ HLA-DR^+^ |  |  |
| **MC15** | HIV-negative | CD20^+^ CXCR4^hi^ CD27^+^ CD24^+^ **CXCR5^hi^** CD4^+^ cMYC^+^  IgM^+^ HLA-DR^+^ | CXCR5 elevated in HIV-negative vs. HIV+ cART-naïve  CXCR5 elevated in HIV-negative vs. HIV+ pre-NHL (cART-naïve) | *p* = 0.001  *p* = 0.013 |
|  | HIV+  cART-naïve | CD20^+^ CXCR4^hi^ CD27^+^ CD24^+^ CXCR5^+^ CD4^+^ cMYC^+^  **IgM^hi^** HLA-DR^+^ | IgM elevated in HIV+ cART-naïve vs. HIV-negative | *p* = 0.032 |
|  | HIV+ pre-NHL (cART-naïve) | CD20^+^ CXCR4^hi^ CD27^+^ CD24^+^ CXCR5^+^ CD4^+^ cMYC^+^  IgM^+^ HLA-DR^+^  EBV LMP1+ |  |  |

**^a^**The 2 CD20^+^CXCR4^hi^ B-cell metaclusters significantly elevated in HIV+ pre-NHL (cART-naïve) compared to HIV+ cART-naïve. Metaclusters are organized by highest to lowest order of significant *p*-values (equal to or greater than -log10 (*p*-value of 0.05) or 1.30) as provided in **Figure 5B**.

**^b^*p*-value:** Significant *p*-value determined when comparing marker expression. Unpaired, non-parametric Mann-Whitney test.

**^c^HIV-negative:** The B-cell phenotypes of each metacluster for HIV-negative are provided in order to compare with the phenotypes observed in HIV+ cART-naïve and HIV+ pre-NHL (cART-naïve) samples.

**^d^**The 4 CD20^+^CXCR4^hi^ B-cell metaclusters significantly elevated in HIV+ cART-naïve compared to HIV+ pre-NHL (cART-naïve). Metaclusters are organized by highest to lowest order of significant *p*-values (equal to or greater than -log10 (*p*-value of 0.05) or 1.30) as provided in **Figure 5B**.

**Supplementary Figures**

**Supplementary Figure 1. CyTOF gating strategy and characterization of viable cells. (A)** Representative gating strategy of PBMCs after CyTOF. Cell-ID Intercalator-Ir (191Ir) vs. time was used to select cells, followed by gating of singlets.194Pt was used to discriminate live and dead cells. **(B)** Gating of CD14^-^CD11b^-^ cells from viable cells and gating of CD19^+^ B-cells from CD14^-^CD11b^-^ cells. **(C)** Contour UMAP plots of viable cells in HIV-negative (n = 10), HIV+ cART-naïve (n = 20), and HIV+ pre-NHL (cART-naïve) (n = 10). UMAPs were created in OMIQ from an equal subsampling of 100,000 cells for each group. **(D)** Representative UMAP plot of viable cells from an HIV-negative sample showing CD19^+^ B-cells, CD19^-^CD20^+^ cells, CD3^+^CD4^+^ T-cells, CD3^+^CD8^+^ T-cells, double positive T-cells (CD3^+^CD4^+^CD8^+^, populations 1 and 2), CD3^+^ double negative T-cells (DN, CD4^-^CD8^-^), CD14^+^ monocytes, CD11b^+^ cells, and other unidentified cell populations (other, population 1 and 2).

**Supplementary Figure 2. Histogram plots of markers in CD19^+^ B-cells.** **(A)** Surface marker expression of CD19, CD14, CD11b, CD3, CD4, and CD8 in CD19^+^ B-cells (CD3^-^CD14^-^CD11b^-^) after CyTOF of PBMCs from HIV-negative (n = 10), HIV+ cART-naïve (n = 20), and HIV+ pre-NHL (cART-naïve) (n = 10) cohort participants. **(B)** Surface marker expression of select classical B-cell activation markers (CD40, CD24, CD38), CD20, B7 family molecules (CD80, CD86, CD27, PD-L1), CD10, CD71, chemokine receptors for signaling (CXCR4, CXCR5), and the B-cell antibody secretion molecule (IgM). Shown are also histograms of intracellular marker expression of the immunoregulatory cytokine IL-10, the transcription factor Bcl-6, and oncogenic markers cMYC and AICDA.

**Supplementary Figure 3**. **Gating strategy of CD19^-^CD20^+^ B-cells. (A-B)** Representative gating strategy of mass cytometry data to identify CD3^-^CD19^-^ cells from gated CD14^-^CD11b^-^ cells (**Supplementary Figure 1A-B**). Data shown are for a representative HIV+ cART-naïve sample (40258-280) and HIV+ pre-NHL (cART-naïve) sample (41602-280). CD19^-^CD20^+^ cells were gated from live CD3^-^CD19^-^ cells. Scatter plots show selection on the CD20 marker in order to discriminate CD20^-^ and CD20^+^ cells (middle and right panels of **A** and **B,** respectively). **(C)** Counts of CD19^-^CD20^+^ B-cells in HIV+ cART-naïve (n = 20) and HIV+ pre-NHL (cART-naïve) (n = 10). Mann-Whitney tests were performed to determine significance: *p*-values ≤ 0.05. ns = non-significant.

**Supplementary Figure 4.** **Metaclusters of** **CD19^-^CD20^+^CXCR4^+^CD24^-^ and CD19^-^CD20^+^CXCR4^+^CD24^+^ B-cells are significantly elevated in HIV+ pre-NHL (cART-naïve), while CD19^-^CD20^+^CXCR4^+^PD-L1^+^BCL-6^+^ B-cells are elevated in HIV+ cART-naïve. (A)** UMAP contour plots of concatenated data from CD19^-^CD20^+^ B-cells in HIV+ cART-naïve (n = 20) and HIV+ pre-NHL (cART-naïve) (n = 10). UMAP plots were generated from an equal subsampling of 20,000 CD19^-^CD20^+^ B-cells for each group. The unsupervised clustering of CD19^-^CD20^+^ B-cells provided 17 metaclusters in HIV+ cART-naïve and HIV+ pre-NHL (cART-naïve) (4 MCs significantly elevated in HIV+ cART-naïve and 2 MCs in HIV+ pre-NHL (cART-naïve). (**B**) Volcano plot showing significantly elevated metaclusters in HIV+ pre-NHL (cART-naïve) and HIV+ cART-naïve. **(C)** Box plots showing cell counts in significantly elevated metaclusters of HIV+ cART-naïve (MC12, MC14, MC15, and MC16) and HIV+ pre-NHL (cART-naïve) (MC01 and MC08) samples. **(D)** Concatenated data summarized in a heatmap with median marker expression values for metaclusters elevated in HIV+ pre-NHL (cART-naïve) (MC01 and MC08). **(E)** Concatenated data summarized in a heatmap with median marker expression values for metaclusters elevated in HIV+ cART-naïve (MC12, MC14, MC15, and MC16).

**Supplementary Figure 5**. **Gating strategy of CD19^+^CD20^+^CXCR4^hi^ B-cells.** **(A-B)** Representative gating strategy of mass cytometry data to identify CD19^+^ B-cells from gated CD14^-^CD11b^-^ cells (**Supplemental Figure 1A-B**). The gating strategy is representative of an HIV+ cART-naïve sample (40258-280) and an HIV+ pre-NHL (cART-naïve) sample (41602-280). CD20^+^CXCR4^+^ and CD20^+^ CXCR4^hi^ cells were gated from viable CD19^+^ B-cells. Scatter plots show selection on the CXCR4 marker in order to gate on CXCR4^hi^ cells (far right panels of **A** and **B,** respectively). **(C)** Counts of CD20^+^CXCR4^+^ B-cells in HIV+ cART-naïve (n = 20) and HIV+ pre-NHL (cART-naïve) (n = 10). **(D)** Counts of CD20^+^CXCR4^hi^ B-cells in HIV+ cART-naïve (n = 20) and HIV+ pre-NHL (cART-naïve) (n = 10). Mann-Whitney tests were performed to determine significance: *p*-values ≤ 0.05. ns = non-significant.

**Supplementary Figure 6**. **Gating strategy of CD19^+^CD24^hi^CD38^hi^ B regulatory cells.** **(A and C)** Representative gating strategy of CD19^+^CD24^hi^CD38^hi^ Bregs from CD19^+^ B-cells (CD3^-^CD14^-^CD11b^-^). The gating strategy is representative of HIV+ cART-naïve (sample 40458-220) **(A)** and HIV+ pre-NHL (cART-naïve) (sample 41849-280) **(C)**. **(B and D)** Representative gating strategies of PD-L1^+^, AICDA^+^, cMYC^+^, IL-10^+^, CD71^+^, FoxP3^+^, BCL-6^+^, IgM^+^, and IgM^hi^ Bregs in HIV+ cART-naïve (sample 40458-220) shown in **A** and HIV+ pre-NHL (cART-naïve) (sample 41849-280) shown in **C**.
